# Photo‐Empowered Macrophage‐Based Drug Delivery System Overcomes Motility Suppression and Significantly Enhances Deep Tumor Drug Delivery

**DOI:** 10.1002/advs.202515349

**Published:** 2025-11-09

**Authors:** Zhaoming Fu, Xin Cui, Shanshan Liu, Lu Gao, Yingang Sun, Yujun Chen, Mianlong Li, Yuxin Fan, Jing Kuang, Wen Song, Feifan Zhou

**Affiliations:** ^1^ State Key Laboratory of Digital Medical Engineering School of Biomedical Engineering Hainan University Sanya 572025 China; ^2^ Key Laboratory of Biomedical Engineering of Hainan Province One Health Institute Hainan University Sanya 572025 China; ^3^ Institute of Pathology Tongji Hospital Tongji Medical College Huazhong University of Science and Technology Wuhan 430030 China

**Keywords:** ATP, deep tumor drug delivery, metabolic suppression, motility, photo‐empowerment

## Abstract

Recently, cell‐based drug delivery systems emerge as highly promising alternatives in the field of antitumor therapy. These systems are characterized by their excellent biocompatibility, robust drug‐loading capacity, and the ability to effectively traverse biological barriers. However, the immunosuppressive tumor microenvironment significantly weakens the motility of immune cells, which is crucial for efficient drug delivery. A novel photo‐empowered macrophage‐based (PEM) drug delivery system is successfully developed in this study. The system is modified with thylakoids extracted from spinach leaves. Upon stimulation with light of specific wavelengths, the thylakoids generate ATP through photochemical reactions, thereby significantly enhancing the motility of macrophages and effectively overcoming the limitations imposed by the tumor microenvironment. In this work, the PEM system is loaded with two antitumor drugs: doxorubicin (Dox) and tirapazamine (TPZ). The results demonstrate that the system not only significantly improves the delivery efficiency of drugs to deep tumor tissues but also greatly enhances antitumor therapeutic efficacy. Particularly after deep infiltration into tumor tissues, the PEM system efficiently eradicates hypoxic and drug‐resistant tumor cells, showcasing remarkable performance. This innovative PEM strategy provides a new approach for deep tumor drug delivery and addressing multidrug resistance, holding great promise for a significant breakthrough in antitumor therapy.

## Graphical abstract

This study develops a photo‐empowered macrophage‐based (PEM) drug delivery system that provides a novel and effective solution for restoring cellular motility to enhance drug delivery and overcome hypoxia‐induced multidrug resistance. The construction of the PEM system fully exploits the unique biological properties of macrophages and the energy‐generating capabilities of thylakoids, drawing inspiration from the process of photosynthesis in plants.

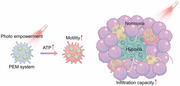

## 1 Introduction

In recent years, significant advantages of cell‐based drug delivery systems (DDS) over traditional DDS have been demonstrated in the field of antitumor drug delivery,^[^ ^1^, ^2^, ^3^ ^]^ making them a research hotspot. Excellent biocompatibility has been shown to enable cell‐based DDS to better adapt to the in vivo environment, significantly reducing the interference caused by immune responses, thereby enhancing the safety and efficacy of drug delivery.^[^ ^4^, ^5^ ^]^ More prominently, the large internal space of cell‐based DDS has been found to endow them with a strong drug‐loading capacity, allowing them to carry a substantial number of drugs.^[^ ^6^, ^7^ ^]^ Additionally, this space provides comprehensive protection for the carried drugs, effectively resisting degradation and clearance by the external environment.^[^ ^8^ ^]^ This ensures the stability and activity of the drugs during delivery, thus improving their therapeutic efficacy. Moreover, the unique biological characteristics of cell‐based DDS enable them to skillfully traverse various biological barriers and precisely deliver drugs to areas that are difficult for traditional DDS to reach.^[^ ^9^, ^10^ ^]^ This significantly enhances the distribution and accumulation efficiency of drugs in target tissues, thereby enhancing their antitumor effects. Building on this foundation, the construction of cell‐based DDS using immune cells with robust tumor‐targeting capabilities,^[^ ^11^, ^12^ ^]^ such as macrophages,^[^ ^13^, ^14^, ^15^ ^]^ is expected to inject a powerful force of innovation into the field of antitumor drug delivery, providing new ideas and strategies for tumor therapy.

However, the immunosuppressive tumor microenvironment significantly impacts immune cells that infiltrate tumor tissues, particularly by interfering with their energy metabolism pathways.^[^ ^16^ ^]^ This interference not only alters the functions and activities of immune cells but also substantially weakens their motility.^[^ ^17^ ^]^ As a result, the efficiency of immune cells in drug delivery may be significantly compromised, especially when they infiltrate deep into the tumor, where they face an extremely hypoxic environment that further restricts their movement. Emerging evidence indicates that exogenous supplementation of adenosine triphosphate (ATP) not only significantly enhances the motility of immune cells but also effectively improves their overall status and functionality.^[^ ^18^, ^19^ ^]^ Based on these findings, we propose that the rational and efficient supplementation of ATP for immune cell‐based DDS holds promise as a pivotal strategy to overcome the bottleneck of the tumor immunosuppressive microenvironment. However, the direct exogenous supplementation of ATP also faces many challenges.^[^ ^20^ ^]^ For example, ATP is prone to hydrolysis in the extracellular environment and body fluids.^[^ ^21^ ^]^ Moreover, there are certain limitations to the uptake of ATP by cells,^[^ ^22^ ^]^ and excessive accumulation of exogenous ATP outside the cells may have toxic effects on the cells.^[^ ^23^ ^]^ Therefore, developing a safe, precise, and efficient ATP supplementation strategy to improve the motility of immune cells and enhance the drug delivery efficiency of immune cell‐based DDS is an urgent problem to be solved.

During the process of plant photosynthesis, thylakoids in chloroplasts can generate nicotinamide adenine dinucleotide under the stimulation of light of specific wavelengths, which is then used for ATP synthesis.^[^ ^24^, ^25^, ^26^ ^]^ Inspired by this, could we construct a photo‐empowered strategy to provide energy support for immune cell‐based DDS? Light, as a clean, non‐invasive, and easily controllable energy source, has unique advantages in this strategy.^[^ ^27^, ^28^, ^29^ ^]^ Specifically, thylakoids could be extracted and used to modify or encapsulate drugs, which are then co‐loaded into immune cells. By irradiating with light of specific wavelengths, the thylakoids inside the cells could be activated, triggering photochemical reactions similar to those in plant photosynthesis, promoting ATP synthesis, enhancing the motility of the cells, and thereby achieving more efficient deep drug delivery.

**Scheme 1:**
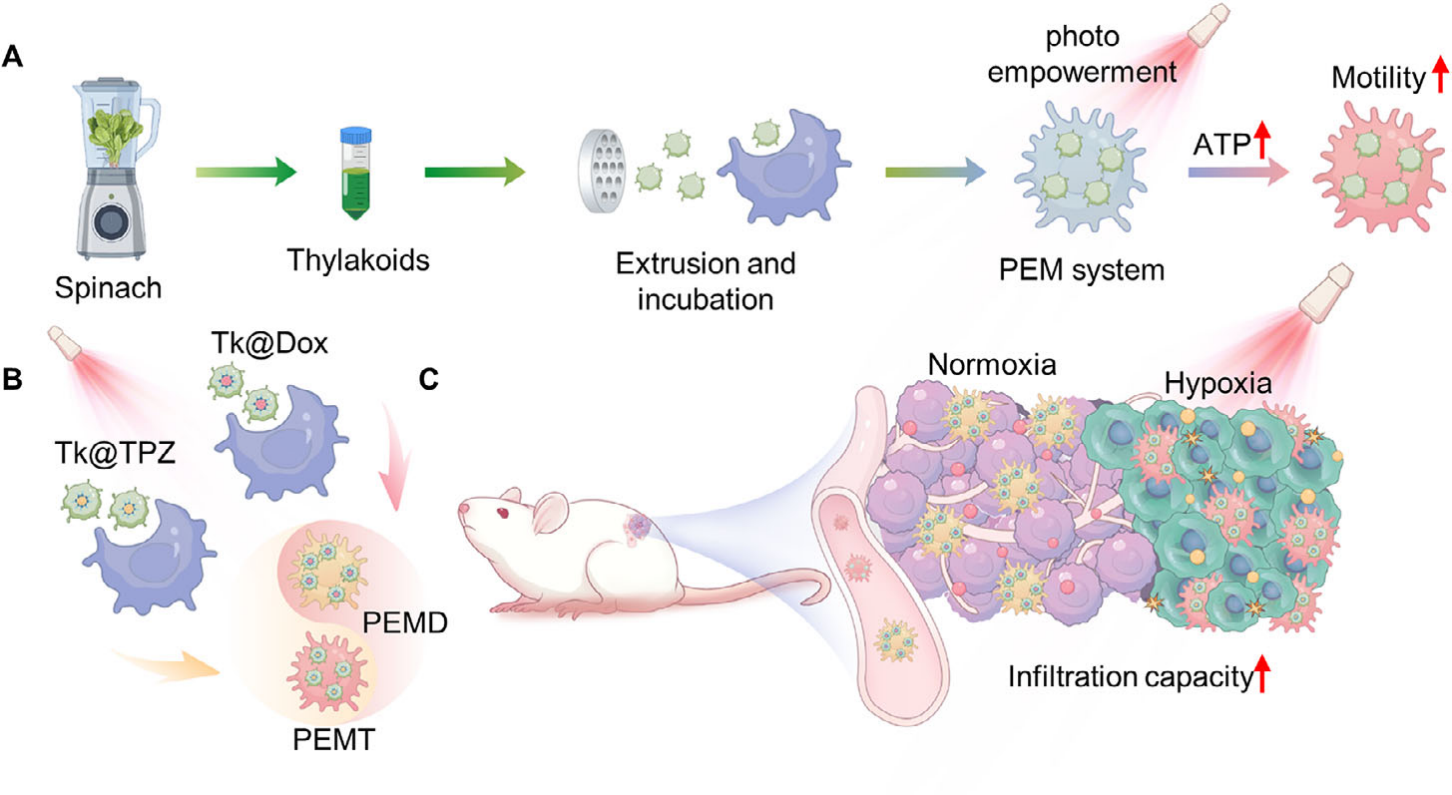
Schematic representation of the photo‐empowered macrophage‐based (PEM) drug delivery system for enhanced antitumor therapy. A) The PEM system is constructed by extracting thylakoids from spinach, processing them through extrusion, and co‐incubating with macrophages. Upon light stimulation, the thylakoids facilitate ATP generation, enhancing macrophage motility and drug delivery efficiency. B) Two drug‐loaded systems, PEMD (doxorubicin‐loaded) and PEMT (tirapazamine‐loaded), are developed. C) In vivo application of PEMD&T in a mouse model demonstrates increased infiltration capacity into both normoxic and hypoxic tumor regions, potentially improving the delivery of Dox and TPZ to enhance antitumor efficacy, particularly targeting hypoxic and drug‐resistant tumor cells. This innovative PEM strategy offers a novel approach for deep drug delivery in the tumor hypoxic microenvironment and addresses multidrug resistance in deep hypoxic tumor cells.

In this study, A photo‐empowered cell‐based DDS based on macrophages was designed and constructed. As shown in **Scheme** **1**A, thylakoids were extracted from spinach leaves and processed through extrusion using a polycarbonate membrane to achieve more uniform and better‐dispersed characteristics.^[^ ^25^, ^26^ ^]^ Subsequently, a photo‐empowered macrophage‐based (PEM) DDS was successfully established by co‐incubating the thylakoids with macrophages. Under light stimulation, ATP generation through photochemical reactions in the thylakoids can provide effective energy support for the PEM system. This process not only effectively overcomes the limitations imposed by the immunosuppressive tumor microenvironment but also significantly enhances the motility of macrophages.

The ability of the PEM strategy to enhance the motility of macrophages through photo‐empowerment was thoroughly investigated, and whether this enhanced motility could promote the infiltration of macrophages into deep tumor regions was further explored. Based on this, two anti‐tumor drugs were selected: doxorubicin (Dox), a traditional chemotherapeutic agent, and tirapazamine (TPZ), a hypoxia‐activated prodrug. These drugs were encapsulated by thylakoids, and further, PEMD and PEMT systems were constructed based on the PEM strategy (Scheme 1B). Using the PEMD&T system as the research object, as shown in Scheme 1C, whether the PEM strategy could promote the deep tumor delivery of Dox and TPZ, thereby enhancing anti‐tumor efficacy, was deeply investigated. Moreover, taking full advantage of the characteristic of TPZ being activated under hypoxic conditions, whether the PEMD&T system could effectively eliminate hypoxic and drug‐resistant tumor cells in the deep tumor regions was further explored. This innovative PEM strategy provides a new approach for anti‐tumor treatment, especially for deep drug delivery targeting the tumor hypoxic microenvironment and addressing multidrug resistance (MDR) in deep hypoxic tumor cells.

## 2 Results and Discussion

### 2.1 PEM Strategy Effectively Overcomes the Bottleneck of the Tumor Immunosuppressive Microenvironment and Significantly Enhances Macrophage Motility

**Figure 1:**
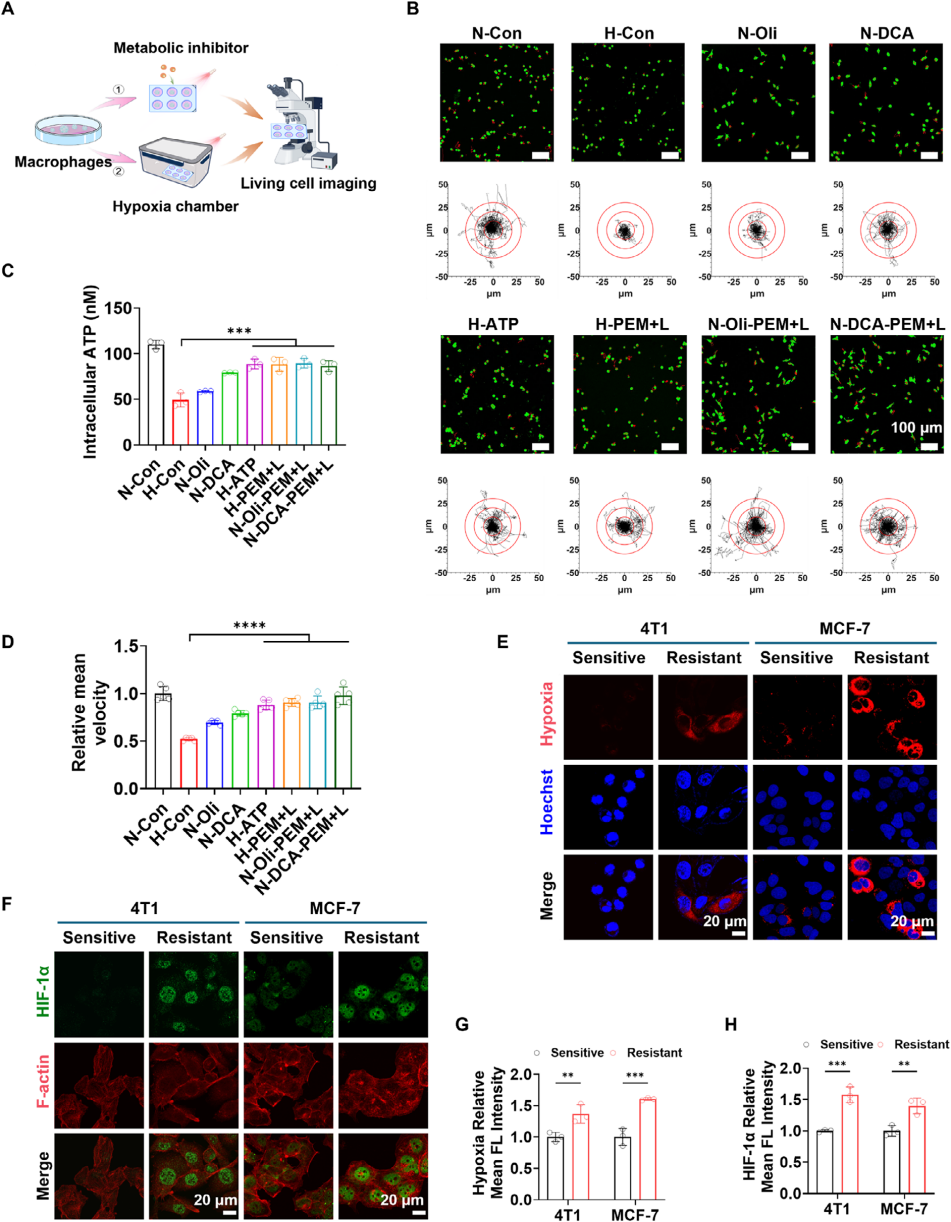
A) Schematic diagram of the experimental design for assessing the motility of Raw264.7 cells after metabolic intervention or hypoxia induction in vitro using living cell imaging technology. B) Representative images and migration tracks of Raw264.7 cells under different treatments. C) Average intracellular ATP content (*n* = 3), and D) cell migration velocity under different treatment conditions (*n* = 5). E) CLSM images of hypoxia levels in 4T1 and MCF‐7 cells marked with hypoxia probes. F) CLSM images of HIF‐1α expression in 4T1 and MCF‐7 cells labeled by immunofluorescence staining. G,H) Semi‐quantitative analysis of hypoxia levels and HIF‐1α expression by fluorescence intensity (*n* = 3). Data are presented as mean ± SD. Statistical significance was calculated via one‐way ANOVA with Tukey’ post hoc test. (*p < 0.05, **p < 0.01, ***p < 0.001, ****p < 0.0001).

Given the objective of employing the PEM strategy to surmount the bottleneck imposed by the tumor immunosuppressive microenvironment and to markedly enhance the motility of macrophages, it is posited that a significant challenge encountered by macrophages during the drug delivery process, as they infiltrate deeper into the tumor, is the progressively intensifying hypoxic environment. To systematically evaluate the impact of hypoxic conditions on the motility of macrophages, a series of in vitro experiments was meticulously designed. As depicted in **Figure** **1**A, macrophages were subjected to various treatments, and their migratory behaviors under different conditions were analyzed utilizing living cell imaging technology. Macrophage motility under normoxic and hypoxic conditions was first compared. As illustrated in Figure 1B, robust motility was observed under normoxic conditions (N‐Con), characterized by extensive and widespread migration trajectories. In contrast, markedly reduced migration paths were detected under hypoxic conditions (H‐Con), indicating that hypoxia may be a key factor impairing macrophage movement. This phenomenon may be closely related to disrupted energy metabolism and insufficient ATP supply under hypoxic conditions, which in turn affects macrophage motility. To test this hypothesis, macrophages were treated with the glycolysis inhibitor Dichloroacetate (DCA) and the ATP synthase inhibitor oligomycin (Oli) under normoxic conditions, and their effects on cell movement were observed. Compared to the N‐Con group, a moderate reduction in motility was observed in macrophages treated with DCA (N‐DCA), while a significant decrease, nearly matching the motility levels observed under hypoxia, was detected in those treated with oligomycin (N‐Oli). These results suggest that both glycolysis and Oxidative phosphorylation (OXPHOS) are essential for maintaining macrophage motility, with OXPHOS playing a more dominant role.

Based on these findings, the hypothesis was proposed that hypoxia impairs macrophage motility by suppressing OXPHOS, thereby reducing ATP production. To further validate this hypothesis, intracellular ATP levels were measured under different treatment conditions. As shown in Figure 1C, the highest ATP levels were detected in the N‐Con group, followed by the N‐DCA group, while similarly low ATP levels were observed in the N‐Oli and H‐Con groups. These results support the hypothesis that hypoxia‐induced suppression of OXPHOS leads to decreased ATP availability, ultimately compromising macrophage motility. Further investigations were conducted to determine whether enhancing intracellular ATP levels could improve macrophage motility under hypoxic conditions. Initially, intracellular ATP content was increased through exogenous ATP supplementation. The results demonstrated that macrophages under a hypoxic environment treated with exogenous ATP (H‐ATP group) exhibited significantly enhanced motility compared to the H‐Con group (Figure 1B), accompanied by a marked increase in intracellular ATP levels (Figure 1C). This suggests that the impaired motility of macrophages under hypoxia can be effectively reversed by ATP supplementation.

Building on this finding, thylakoid membranes were extracted from spinach and co‐incubated with macrophages to develop a PEM‐based drug delivery system. It was hypothesized that thylakoid‐mediated photochemical reactions could generate ATP within the cells, thereby compensating for the ATP deficit caused by suppressed OXPHOS or glycolysis. The experimental results demonstrated that under hypoxic conditions, macrophages in the H‐PEM+L group showed significantly improved motility and increased intracellular ATP levels compared to the H‐Con group (Figure 1B,C). This further confirms that the PEM strategy can effectively elevate intracellular ATP levels and restore macrophage motility impaired by hypoxia. The investigation was then extended to evaluate the effects of the PEM strategy on macrophage motility under normoxic conditions, particularly in the presence of metabolic inhibitors (N‐Oli and N‐DCA groups). The results revealed that macrophages in the N‐Oli‐PEM+L and N‐DCA‐PEM+L groups exhibited significantly enhanced motility compared to their respective control groups (N‐Oli and N‐DCA), with corresponding increases in intracellular ATP levels (Figure 1C). These findings further underscore the close relationship between macrophage motility and intracellular ATP availability, and highlight that hypoxia‐induced metabolic disruption leads to ATP depletion. The PEM strategy effectively replenishes intracellular ATP, thereby restoring macrophage motility.

Finally, living cell imaging data were analyzed to quantify macrophage motility under different treatment conditions. Cell trajectory analysis was used to calculate motility speed scores. As shown in Figure 1D, hypoxia and metabolic inhibitor treatments significantly reduced macrophage motility speed scores compared to normoxic conditions. In contrast, both the PEM strategy and exogenous ATP supplementation markedly improved macrophage motility under hypoxic conditions. These results confirm that hypoxia is a key factor contributing to the decline in macrophage motility, and that the PEM strategy represents an effective intervention to restore macrophage function under such conditions.

### 2.2 The Association between Multidrug Resistance and Hypoxia

The hypoxic state of tumors has been identified as a significant factor influencing the activity and function of immune cells and is also one of the key contributors to tumor MDR.^[^ ^30^, ^31^ ^]^ Studies have demonstrated that under hypoxic conditions, hypoxia‐inducible factor‐1 (HIF‐1) can enhance cellular responses to DNA damage, thereby helping to maintain genomic stability. Additionally, it has been found that the activation of HIF‐1 can reduce the expression levels of topoisomerase II in cancer cells, which may diminish the DNA‐damaging effects of chemotherapeutic drugs and thus promote the development of MDR.^[^ ^32^ ^]^ Based on these findings, a PEM strategy to enhance macrophage motility has been proposed. This strategy holds potential for improving the efficiency of drug delivery to deep‐seated tumors, which has been shown to significantly enhance anti‐tumor therapeutic efficacy.^[^ ^33^, ^34^, ^35^ ^]^ Additionally, investigations are planned to explore whether this PEM strategy can augment the therapeutic efficacy against drug‐resistant tumor cells. Consequently, further investigation has been conducted into the underlying mechanisms linking tumor MDR and hypoxia.

To investigate the intrinsic relationship between tumor drug resistance and hypoxia, two cell lines, 4T1 and MCF‐7, were selected, and Dox‐resistant sublines were established. The hypoxic status of each cell line was systematically assessed using a hypoxia probe. As shown in Figure 1E,G, strong red fluorescence signals were detected in the drug‐resistant cell lines, indicating a significant hypoxic state. In contrast, no such fluorescence was observed in the Dox‐sensitive cell lines. This marked difference suggests that hypoxia levels are significantly elevated in drug‐resistant tumor cells compared to sensitive cells, implying a potential critical role of hypoxia in the mechanisms underlying tumor cell resistance. Building upon the initial hypoxia assessment across different cell lines, immunofluorescence staining was further employed to evaluate the expression levels of HIF‐1α in both sensitive and resistant cell lines. HIF‐1α, a key regulatory factor in the cellular response to hypoxia, plays a critical role in cellular adaptation to low‐oxygen environments. As shown in Figure 1F,H, significantly elevated expression levels of HIF‐1α were observed in the resistant cell lines compared to the sensitive ones, as clearly indicated by the intense fluorescence signals. HIF‐1α not only serves as a biomarker of hypoxia but also participates in the transcriptional regulation of genes involved in angiogenesis and metabolic reprogramming. These biological processes are likely to contribute directly to the development of chemoresistance in tumor cells.^[^ ^32^ ^]^ Therefore, the high expression of HIF‐1α in the resistant cell lines further supports a strong association between hypoxic status and chemotherapeutic resistance.

In addition, immunofluorescence staining was also employed to evaluate the expression of Xeroderma Pigmentosum, Complementation Group F (XPF) protein in the MCF‐7 cell line. XPF plays a critical role in DNA damage repair and can significantly enhance the ability of tumor cells to repair DNA lesions, thereby potentially reducing the therapeutic efficacy of DNA‐targeting chemotherapeutic agents such as doxorubicin and platinum‐based drugs. Therefore, the expression level of XPF protein serves as an important indicator for assessing tumor cell resistance. As shown in Figure S1 (Supporting Information), markedly higher intensity of green fluorescence was observed in the resistant cell lines compared to the sensitive ones, indicating a significant upregulation of XPF protein expression in resistant tumor cells.

### 2.3 Investigating the Relationship between Macrophage Motility and the Efficiency of Deep Drug Delivery In Vitro

**Figure 2:**
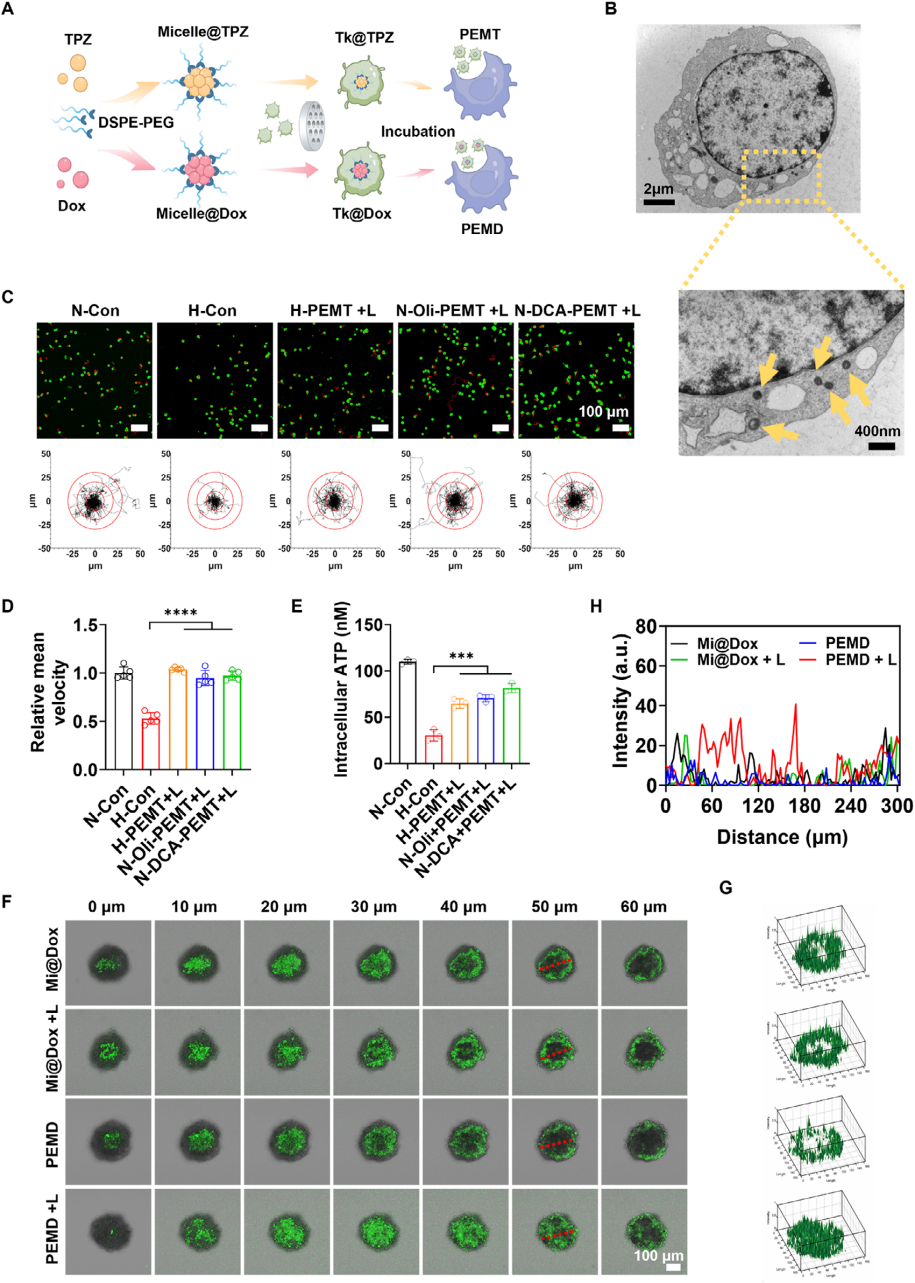
A) Schematic illustration of the construction processes of PEMD and PEMT. B) Bio‐TEM images of PEMD&T. The yellow dotted box indicates the area of local magnification, and the yellow arrows point to PEMD&T. C) Representative images and migration tracks of Raw264.7 cells under different treatment conditions, as well as the D) mean migration velocity (*n* = 5) and E) Average intracellular ATP content of Raw264.7 cells (*n* = 3). F) Penetration of PEMD and Mi@Dox into 3D tumor spheroids at different depths under light or dark conditions. G) 3D reconstruction showing the penetration of PEMD and Mi@Dox at a depth of 50 µm within the tumor spheroid (with a Z‐stack thickness of 10 µm). H) Quantitative analysis of fluorescence intensity along the red‐marked line in the 50‐µm depth region of the tumor spheroid. Data are presented as mean ± SD. Statistical significance was calculated via one‐way ANOVA with Tukey’ post hoc test. (*p < 0.05, **p < 0.01, ***p < 0.001, ****p < 0.0001).

After confirming that the PEM strategy could significantly enhance macrophage motility, the question arose as to whether this locomotor gain could translate into more efficient deep‐tissue drug delivery. As illustrated in **Figure** **2**A, Dox and TPZ were separately encapsulated in DSPE‐PEG micelles, onto which spinach‐derived thylakoids were anchored via the carbonate membrane extrusion method (detailed physicochemical data are provided in the Figures S2–S5, Supporting Information). Subsequent co‐incubation with macrophages developed the PEMD&T system.

Bio‐transmission electron microscopy (Figure 2B) revealed widespread cytoplasmic distribution of ≈100 nm micelles, confirming efficient PEMD&T internalization. Drug‐loading efficiencies and release kinetics are also supplied in Figures S6 and S7 (Supporting Information). Using TPZ‐loaded PEMT as a model, living cell imaging assessed its motility under varied conditions. As illustrated in Figure 2C, under hypoxic conditions, the motility trajectory of the H‐PEMT+L group was significantly superior to that of the H‐Con group, and its motility speed score nearly matched that of the normoxic group (N‐Con group) (Figure 2D). Under normoxia, even after metabolic suppression with oligomycin or DCA, the motility speed score of PEMT+L macrophages (N‐Oli‐PEMT+L and N‐DCA‐PEMT+L groups) remained significantly above the H‐Con value and approached the N‐Con level (Figure 2D). These findings indicate that TPZ loading does not compromise the light‐empowering effect of the PEM system. Coupled with intracellular ATP quantification, the conclusion is that PEMT, through photosynthetic empowerment, reverses motility deficits imposed by hypoxia or metabolic inhibition (Figure 2E).

The focus was then placed on PEMD as the subject of the study, with the intrinsic fluorescence of Dox being capitalized on. The layer‐by‐layer scanning technique of confocal laser microscopy was employed to meticulously examine the distribution differences of Dox at various depths within 3D tumor spheroids. As is illustrated in Figure 2F, within the depth range of 0–60 µm, the fluorescence signal distribution between the pure drug‐loaded micelles (Mi@Dox) group and the light‐irradiated group (Mi@Dox+L) was nearly indistinguishable and could be considered negligible. In contrast, a striking difference in fluorescence signal distribution was observed between the PEMD group and the PEMD+L group. The light treatment significantly enhanced the penetration of Dox into deeper regions of the tumor spheroids, indicating that light played a pivotal role in promoting PEMD‐mediated drug delivery. 3D reconstruction analysis of the fluorescence signals at a depth of 50 µm was conducted (Figure 2G). The results revealed that, except for the PEMD+L group, the Dox fluorescence signals of the other three groups were predominantly confined to the peripheral regions of the spheroids. However, the Dox fluorescence signal in the PEMD+L group penetrated into the core of the spheroid. This finding clearly demonstrates that the PEM strategy, through light‐activated macrophage motility, achieves more efficient and deeper drug delivery. The quantitative analysis of the fluorescence distribution in Figure 2H further corroborates this conclusion.

### 2.4 Assessing the Promoting Effects of PEM Strategy on Macrophage Motility and Deep Tissue Infiltration In Vivo Using Dorsal Window Intravital Imaging Technology

**Figure 3:**
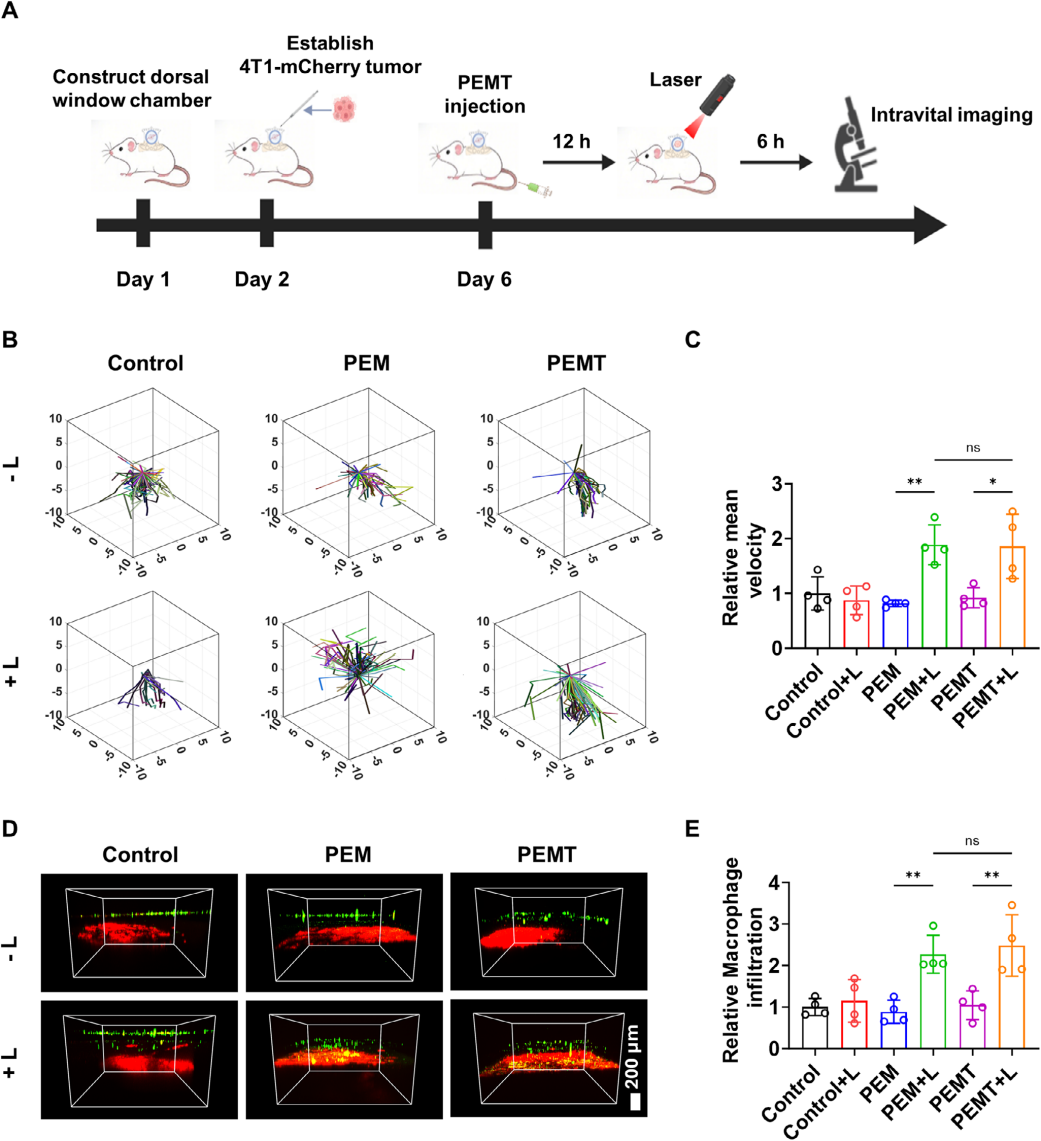
A) Schematic illustration of intravital imaging experiments using a dorsal window chamber to observe CMAC‐labeled Raw264.7 cells within 4T1‐mCherry tumors. B) Representative 3D migration tracks of Raw264.7 cells within the 4T1 tumor region under different conditions, and C) mean migration velocity of Raw264.7 cells (*n* = 4). D) Representative intravital imaging of CMAC‐labeled Raw264.7 cell infiltration into tumor tissues (red fluorescence indicates tumor cells, green fluorescence indicates Raw264.7 cells). E) Quantitative fluorescence analysis of macrophage infiltration into tumor regions (*n* = 4). Data are presented as mean ± SD. Statistical significance was calculated via one‐way ANOVA with Tukey’ post hoc test. (*p < 0.05, **p < 0.01, ***p < 0.001).

The effects of the PEM strategy on the motility of macrophages and their deep tumor infiltration capacity in vivo were investigated thoroughly using intravital imaging techniques. As shown in **Figure** **3**A, a dorsal skinfold window model was established in mice first, and an mCherry‐labeled 4T1 tumor model was constructed the following day. Different macrophages labeled with CellTracker Blue (CMAC) were introduced into the mice via intravenous injection on the sixth day. Subsequently, the mice were randomly divided into two groups: one group received light irradiation on the dorsal skin 6 h after injection, while the other group remained untreated. 12 h post‐injection, a confocal laser scanning microscope (CLSM) was employed to observe the movement and infiltration of macrophages over an extended period.

The motility range of macrophages in the Control group (macrophages without any treatment) was relatively small, with no significant differences observed between the light‐irradiated and non‐irradiated conditions (Figure 3B). This indicated that the motility of normal macrophages was restricted in the tumor immunosuppressive microenvironment. In contrast, the motility range of macrophages in the PEM group exhibited markedly different under light‐irradiated and non‐irradiated conditions, highlighting a significant difference between the two scenarios. Specifically, the motility range of the PEM group was similar to that of the Control group in the absence of light irradiation. However, under light irradiation, the motility range of the macrophages in the PEM group dramatically increased, and their motility performance was significantly enhanced. Similar results and trends were observed in the PEMT and PEMT+L groups, which further confirmed that the PEM strategy could significantly enhance the in vivo motility of macrophages even after drug loading.

The movement trajectories of the macrophages were processed and analyzed to evaluate their motility speed under different conditions. As illustrated in Figure 3C, the highest motility speed was exhibited by the macrophages in the PEM+L and PEMT+L groups, which was significantly higher than that of the non‐irradiated PEM and PEMT groups and the Control group. The tumor infiltration capacity of different macrophages was assessed using intravital imaging techniques. As shown in Figure 3D, in the Control group, regardless of light irradiation, the green fluorescence representing macrophages was distributed in the peripheral region of the red fluorescence representing tumor cells, indicating that normal macrophages had insufficient ability to infiltrate tumor tissues, even under light stimulation. Similarly, in the non‐irradiated conditions, the PEM and PEMT groups showed similar results. However, under light irradiation, a large amount of green fluorescence was observed in the interior and central regions of the red fluorescence in the PEM and PEMT groups, which fully demonstrated that the PEM strategy could significantly promote the deep tumor infiltration capacity of macrophages in vivo. The relative infiltration index of macrophages was calculated through further analysis of the results shown in Figure 3D. As illustrated in Figure 3E, the indices in the PEM+L and PEMT+L groups were comparable and significantly higher than those in the other four groups, nearly doubling the latter. These findings further indicate that the PEM strategy can significantly enhance the tumor infiltration efficiency of macrophages.

### 2.5 In Vivo Tumor Targeting and Deep Drug Delivery of PEM System

**Figure 4:**
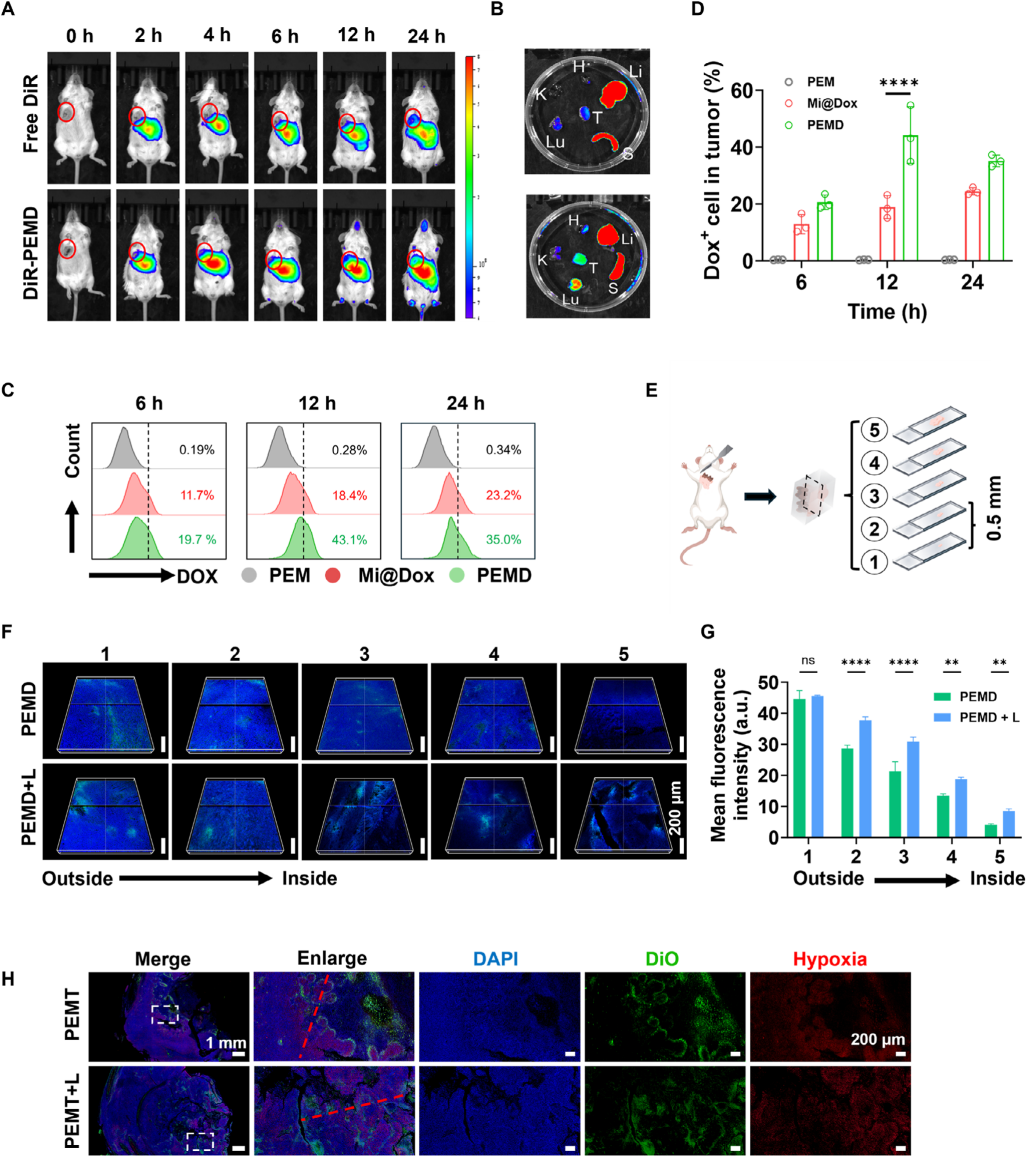
A) In vivo fluorescence imaging of the distribution of Free DiR and DiR‐PEMD in mice at different time points following intravenous injection. B) In vivo imaging of fluorescence distribution in major mouse tissues (heart, liver, spleen, lung, and kidney) 24 h post‐intravenous injection. C) Flow cytometric analysis of drug distribution in tumor tissues at different time points after intravenous injection of PEM, Mi@DOX, and PEMD, and D) quantitative analysis of fluorescence intensity (*n* = 3). E) Schematic illustration of the experimental design for sectioning and staining of mouse tumor tissues 12 h post‐intravenous injection to observe drug penetration. F) Representative CLSM images of tumor sections at different depths (green fluorescence indicates Dox), and G) semi‐quantitative analysis of Dox fluorescence intensity in sections at different depths (*n* = 3). H) CLSM images of DiO‐labeled PEMT (green fluorescence) and pimonidazole‐labeled hypoxic regions (red fluorescence). Data are presented as mean ± SD. Statistical significance was calculated via one‐way ANOVA with Tukey’ post hoc test and two‐way ANOVA with the Scheffé post hoc test. (*p < 0.05, **p < 0.01, ***p < 0.001, ****p < 0.0001).

After verifying that the PEM strategy could significantly enhance the motility of macrophages and their infiltration into the deep layers of tumors, the tumor‐targeting performance and deep drug delivery capabilities of the PEM system were further investigated. Initially, macrophages were labeled with the membrane dye DiR and introduced into mice via intravenous injection. Subsequently, the tumor‐targeting performance of PEMD was monitored in real time using an animal fluorescence imaging system. As shown in **Figure** **4**A, over time, DiR‐labeled PEMD gradually accumulated in the tumor tissue, exhibiting a distinct targeting trend. Furthermore, the fluorescence intensity of PEMD in the tumor region was quantitatively analyzed at different time points (Figure S8, Supporting Information). Compared with the free DiR group, PEMD was found to have significantly higher fluorescence intensity at 12 h post‐injection, which fully demonstrated that the PEM system could achieve precise tumor targeting by leveraging the inherent tumor‐homing ability of macrophages. Twenty‐four hours after intravenous injection, the major organs of the mice were excised, and fluorescence imaging was performed (Figure 4B). Compared with the free DiR group, PEMD maintained a strong fluorescence signal in the tumor tissue (with the specific semi‐quantitative fluorescence values shown in Figure S9, Supporting Information). This observation not only confirms the excellent tumor‐targeting capability of the PEM system but also indicates its favorable retention in the tumor tissue, thereby establishing a robust foundation for subsequent drug release.

Additionally, micelles encapsulating Dox (Mi@Dox) and a drug‐free PEM system were introduced as control groups to further validate the tumor‐targeting efficacy of PEMD. The tumor tissue was excised at different time points after intravenous injection of PEMD, PEM, and Mi@Dox, and then homogenized into a single‐cell suspension for subsequent detection and analysis by flow cytometry. As shown in Figure 4C,D, at 6, 12, and 24 h after intravenous injection, the Dox content detected in the tumor tissue in the PEMD group was significantly higher than that in the Mi@Dox group. Particularly, at 12 h post‐intravenous injection, the concentration of Dox in tumor tissues of the PEMD group reached 43.1%, which was significantly higher than that of the Mi@Dox group (18.4%). Meanwhile, no significant fluorescence signal was detected in the tumor single‐cell suspensions of the PEM group at 6, 12, and 24 h under excitation at 488 nm, which fully ruled out any potential fluorescence interference from the PEM system itself. This result further highlighted the significant advantage of the macrophage‐based drug delivery system over traditional drug delivery systems in terms of tumor targeting.

After verifying the excellent tumor‐targeting ability of the PEM system, whether the ability of the PEM system to deliver drugs to deeper regions of the tumor could be enhanced by light irradiation was further explored. As shown in Figure 4E, based on the results of Figure S8 (Supporting Information) and Figure 4D, mice that had been intravenously injected with PEMD for 12 h were randomly divided into two groups: one group received 5 min of light irradiation, while the other group received no treatment. After 24 h of intravenous injection, the tumor tissues were harvested from the mice and sectioned from the outside to the inside at intervals of 0.5 mm. Subsequently, the fluorescence of Dox was observed using a CLSM. As depicted in Figure 4F, both the light‐irradiated and non‐irradiated groups exhibited bright green fluorescence, indicative of the presence of Dox, in the outermost layer (first layer) of the tumor tissue. However, starting from the second layer, the green fluorescence in the tumor sections of the PEMD group progressively diminished, whereas the PEMD+L group maintained a strong fluorescence signal. Notably, in the fifth layer, the green fluorescence representing Dox was virtually undetectable in the PEMD group, while it remained clearly visible in the PEMD+L group. In conjunction with the semi‐quantitative analysis of fluorescence intensity across different layers (Figure 4G), these observations clearly demonstrate that the PEM strategy significantly enhances the delivery of drugs to deeper tumor regions, with light irradiation playing a crucial role in this process.

Next, the drug loaded by PEM was replaced with the hypoxia‐activated prodrug TPZ, and PEMT was fluorescently labeled with the cell membrane dye DiO. After intravenously injecting PEMT into the mice, the experimental methodology of Figure 4F was followed, and the hypoxic regions in the tumor tissues were specifically marked with pimonidazole before harvesting the tumor tissues. Subsequently, the tumor tissues were sectioned and subjected to immunofluorescence staining according to the depth standard of the fifth layer in Figure 4F. As shown in Figure 4H, in the absence of light irradiation, the green fluorescence of DiO‐labeled PEMT was mainly distributed in the periphery of the red fluorescence representing the hypoxic regions, indicating that PEMT had difficulty penetrating into the deeper hypoxic regions of the tumor without light stimulation. However, in the PEMT+L group, under light irradiation, the green fluorescence largely overlapped with the red fluorescence, demonstrating that light irradiation significantly enhanced the infiltration of PEMT into the deeper hypoxic regions of the tumor, enabling it to effectively reach the hypoxic sites in the tumor's deeper layers. This finding is of great significance for the precise delivery of the hypoxia‐activated prodrug TPZ to the deeper hypoxic regions of the tumor by the PEM system. Further processing and analysis of Figure 4H, including semi‐quantitative analysis of the fluorescence intensity in the red‐lined regions, yielded results as shown in Figure S10 (Supporting Information). Under non‐irradiated conditions, minimal overlap was observed between the green lines representing PEMT and the red lines representing hypoxic regions. In contrast, substantial overlap between the green and red lines was evident in the PEMT+L group. These findings further demonstrate that light irradiation significantly promotes the penetration of PEMT into the deep hypoxic regions of the tumor.

### 2.6 PEM System for In Vivo Orthotopic Treatment of TNBC

**Figure 5:**
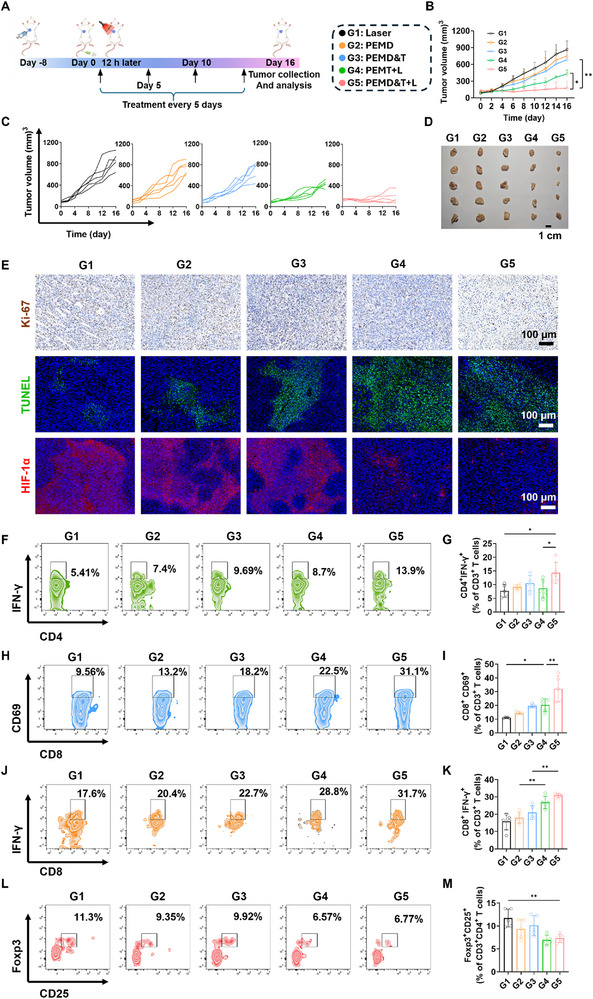
A) Schematic illustration of the 16‐day treatment of the PEM system in 4T1 tumor‐bearing mice. B) Tumor volume changes over time in different treatment groups, and C) in individual mice (*n* = 5). D) Photograph of tumor tissues from different treatment groups after 16 days of treatment (*n* = 5). E) Histopathological sections of tumor tissues stained with Ki‐67, TUNEL, and HIF‐1α after 16 days of treatment. Flow cytometric analysis of tumor‐infiltrating F) CD4^+^IFN^+^ T cells, H) CD8^+^CD69^+^ T cells, J) CD8^+^IFN^+^ T cells, and L) Tregs in tumor tissues after 16 days of treatment, along with the corresponding statistical data (G,I,K,M) (*n* = 5). Data are presented as mean ± SD. Statistical significance was calculated via one‐way ANOVA with Tukey’ post hoc test. (*p < 0.05, **p < 0.01, ***p < 0.001).

The in vivo anticancer efficacy of the PEM system was investigated using a mouse model of triple‐negative breast cancer (TNBC). As shown in **Figure** **5**A, during the 16‐day treatment period, PEMD&T was administered via intravenous injection on days 0, 5, 10, and 15, followed by light irradiation of the tumor region 12 h post‐administration. Upon completion of the treatment, the tumor growth curves of the mice in each group were analyzed (Figure 5B,C), the tumor tissues were photographed (Figure 5D), and the tumor weights of each group were recorded (Figure S11, Supporting Information). The results showed that the PEMD&T+L group exhibited the most significant tumor suppression, with the smallest tumor volume and a significantly reduced tumor growth rate. Compared with the PEMD&T+L group, the PEMT+L group had slightly inferior antitumor effects but still significantly outperformed the other three groups. These findings indicate that the PEM strategy can significantly improve the motility of macrophages, thereby enhancing drug delivery efficiency and significantly boosting antitumor efficacy. Moreover, the combination of Dox and TPZ exhibited better tumor inhibition than TPZ alone. During the treatment, the body weight changes of the mice in each group were closely monitored. As shown in Figure S12 (Supporting Information), there were no significant fluctuations in the body weight of the mice throughout the treatment period, indicating that the treatment did not have a significant negative impact on the overall physiological state of the mice. Additionally, upon completion of the treatment, hematoxylin‐eosin (H&E) staining was performed on the major organs of the mice in each group (Figure S13, Supporting Information). The results showed no obvious pathological changes in the major organs of the mice in any group. This further confirms the safety of the PEM system for in vivo drug delivery.

The tumor tissues of the mice in each group were sectioned and subjected to H&E, immunohistochemical, and immunofluorescence staining after the treatment. As shown in Figure 5E, consistent with the trend of treatment efficacy, the tumor sections of the G5 group had the lowest expression of Ki‐67, a marker closely related to cell proliferation, and the most specific fluorescence in TUNEL staining, which is closely related to cell apoptosis. This trend was also verified in the H&E staining results (Figure S14, Supporting Information). Meanwhile, immunofluorescence staining for HIF‐α expression in the tumor tissues revealed that the HIF‐α expression was lowest in the G4 and G5 groups. This indicates that the PEM strategy, by delivering hypoxia‐activated prodrugs such as TPZ deep into the tumor tissue, can significantly improve the hypoxic condition of the tumor tissue. Moreover, in combination with traditional chemotherapeutic drugs such as Dox, it can further enhance the therapeutic effect.

The detailed phenotypic analysis of immune cells in tumor tissues was performed using flow cytometry to thoroughly investigate whether the PEM system can effectively activate antitumor immune responses. The activation degree of helper T cells was assessed by detecting the expression levels of IFN‐γ in CD3^+^CD4^+^ T cells. As is well known, the adequate activation of helper T cells is crucial for enhancing the cytotoxic function of CD8^+^ T cells and is a key step in achieving effective antitumor immune responses.^[^ ^36^ ^]^ As shown in Figure 5F,G, the proportion of activated helper T cells in the tumor tissues of the G5 group was found to be significantly higher than that in the other groups. This indicates that the PEM strategy can efficiently activate helper T cells, providing strong support for the subsequent activation and functional exertion of CD8^+^ T cells.

The activation state and effector functions of CD8^+^ T cells were further explored. The expression of CD69 and IFN‐γ in CD3^+^CD8^+^ cells was analyzed, and it was found that, compared with the control group (G1), the proportion of CD3^+^CD8^+^CD69^+^ cells in the tumor tissues of the other groups increased significantly, as shown in Figure 5H,I. This suggests that both Dox and TPZ can activate CD8^+^ T cells to some extent. The activation effect was further enhanced by the application of the PEM system, with the most significant activation observed in the G5 group. Correspondingly, the proportion of CD3^+^CD8^+^IFN‐γ^+^ cells in the tumor tissues of the G5 group was also significantly higher than that in the other four groups, as shown in Figure 5J,K. This indicates that improving drug delivery efficiency not only effectively enhanced the activation level of CD8^+^ T cells but also significantly improved their effector functions, thereby significantly enhancing the cytotoxic function of CD8^+^ T cells and achieving a stronger antitumor effect.

The proportion of regulatory T cells (Tregs) in the tumor tissues was assessed by detecting the expression of CD25 and FOXP3 in CD3^+^CD4^+^ cells. Tregs exert immunosuppressive effects in the tumor microenvironment, and a reduced proportion of Tregs implies a weakened immunosuppressive effect of the tumor immune microenvironment on immune cells, which is conducive to enhancing the antitumor activity of immune cells.^[^ ^37^ ^]^ As shown in Figure 5L,M, the proportion of Tregs in the tumor tissues of the G5 group was found to be significantly lower, decreasing from 11.3% in the control group (G1) to 6.77% in the G5 group. This further confirms that the PEMD&T+L treatment strategy can effectively improve the immunosuppressive tumor microenvironment.

In summary, the drug delivery efficiency of Dox and TPZ was significantly enhanced through light‐empowered activation by the PEM strategy, thereby significantly enhancing the activity of immune cells in tumor tissues and effectively improving the immunosuppressive tumor microenvironment. This series of synergistic mechanisms ultimately achieved efficient treatment of TNBC.

### 2.7 The PEM System Activates Peripheral Immune Cells

**Figure 6:**
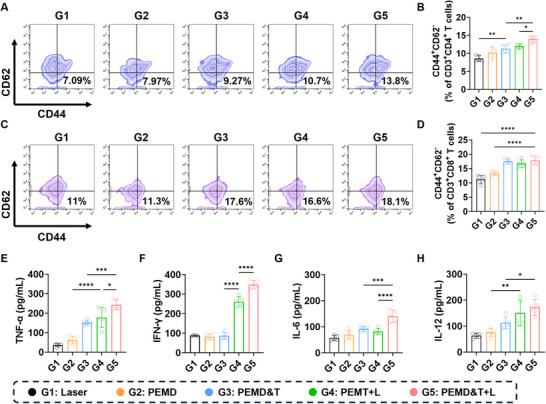
Flow cytometric analysis of A) CD4^+^ effector memory T cells and C) CD8^+^ effector memory T cells in the spleens of mice from different groups after 16 days of treatment, along with the corresponding quantitative data (B,D) (*n *= 5). Concentrations of cytokines in the peripheral blood serum of mice from different groups after 16 days of treatment, as determined by ELISA: E) TNF‐α, F) INF‐γ, G) IL‐6, and H) IL‐12 (*n* = 5). Data are presented as mean ± SD. Statistical significance was calculated via one‐way ANOVA with Tukey’ post hoc test. (*p < 0.05, **p < 0.01, ***p < 0.001, ****p < 0.0001).

After the treatment was completed, the effect of the PEM system on the peripheral immune system of mice was further investigated. The proportion of CD4^+^ effector T cells and CD8^+^ effector T cells in the spleen of mice was detected using flow cytometry. As shown in **Figure** **6**A,C, a significant increase was observed in the proportions of CD4⁺ and CD8^+^ effector T cells in the spleen of mice in the G5 Group. Specifically, the proportion of CD3^+^CD4⁺CD44⁺CD62^−^ effector T cells increased from 7.09% in G1 Group to 13.8% in G5 Group, while the proportion of CD3^+^CD8⁺CD44⁺CD62^−^ effector T cells rose from 11% in G1 Group to 18.1% in G5 Group. These results indicate that the treatment in G5 Group significantly activated the peripheral immune system of mice, promoting the proliferation and differentiation of naive T cells into effector T cells under the influence of antigen stimulation and other factors.

The secretion of various cytokines in the serum of mice from different groups was detected using ELISA. As shown in Figure 6E,F, the expression levels of TNF‐α and IFN‐γ in the G5 Group were significantly elevated, which further confirmed the activated state of the immune system in the G5 group. The secretion of TNF‐α and IFN‐γ can enhance the activity of immune cells such as macrophages and T cells, thereby promoting the killing and elimination of tumor cells by immune cells. In addition, as shown in Figure 6G,H, the expression levels of IL‐6 and IL‐12 were also significantly increased in the G5 Group. IL‐6 can participate in inflammatory responses and regulate the differentiation of T cells, while IL‐12 can induce immune cells to produce IFN‐γ, promote the differentiation of Th1 cells, and further enhance the cellular immune response.

Overall, clear signs of immune activation, observed in both T cell differentiation and cytokine secretion, indicate that the PEM strategy had a significant impact on the peripheral immune system of mice.

### 2.8 PEM System for In Vivo Orthotopic Treatment of Dox‐Resistant TNBC

**Figure 7:**
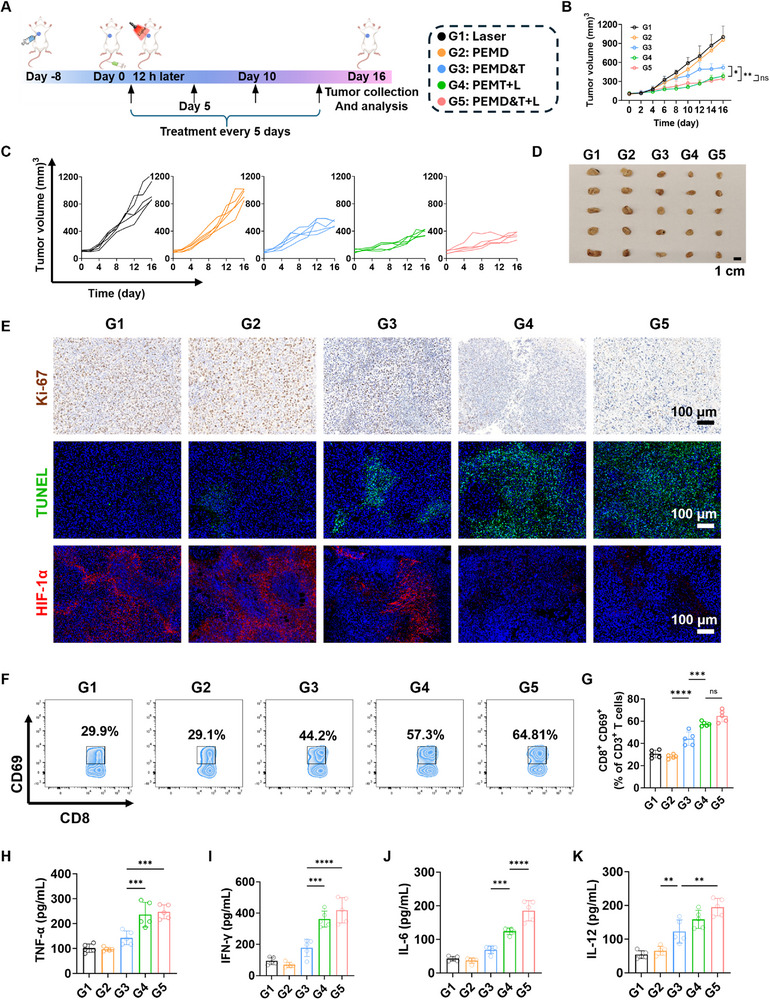
A) Schematic illustration of the 16‐day treatment protocol of the PEM system in 4T1‐ADR tumor‐bearing mice. B) Tumor volume changes over time in different treatment groups, and C) in individual mice (*n* = 5). D) Photograph of tumor tissues from different treatment groups after 16 days of treatment (*n* = 5). E) Histopathological sections of tumor tissues stained with Ki‐67, TUNEL, and HIF‐1α after 16 days of treatment. F) Flow cytometric analysis of tumor‐infiltrating CD8^+^CD69^+^ T cells in tumor tissues after 16 days of treatment, along with the corresponding statistical data (G) (*n* = 5). Concentrations of cytokines in the peripheral blood serum of mice from different groups after 16 days of treatment, as determined by ELISA: H) TNF‐α, I) INF‐γ, J) IL‐6, and K) IL‐12 (*n *= 5). Data are presented as mean ± SD. Statistical significance was calculated via one‐way ANOVA with Tukey’ post hoc test. (*p < 0.05, **p < 0.01, ***p < 0.001, ****p < 0.0001).

A drug‐resistant tumor model was established using 4T1‐ADR cells, which are resistant to doxorubicin, and the antitumor efficacy of the PEM system was investigated under the same experimental conditions (**Figure** **7**A). Following a 16‐day treatment period, the tumor size in mice was evaluated. As shown in Figure 7B,C, no significant difference in tumor growth inhibition was observed between G1 and G2. These results indicate that even when the PEM system, which has excellent tumor‐targeting capabilities, is used as a drug delivery vehicle for Dox, the therapeutic effect on Dox‐resistant tumors is not enhanced. This suggests that simply improving drug delivery efficiency cannot enhance the inhibitory effect of Dox on drug‐resistant cells. In comparison, the PEMD&T group exhibited a certain degree of tumor inhibitory effect, which further confirmed that the PEM system can deliver TPZ to the hypoxic regions of the tumor to some extent. Moreover, the tumor growth inhibitory effects of the PEMT+L and PEMD&T+L groups were more pronounced than those of the non‐irradiated groups. These findings indicate that under light irradiation, the PEM system can more deeply penetrate the hypoxic regions of the tumor and fully exert the hypoxia‐activated properties of TPZ, thereby significantly enhancing the antitumor effect.

Tumor images and weights for different treatment groups are shown in Figure 7D and Figure S15 (Supporting Information). Mouse body weight changes during the 16‐day treatment period are presented in Figure S16 (Supporting Information). Following the treatment, immunohistochemical and immunofluorescence staining were performed on the tumor tissues from the mice. The results (Figure 7E) show that the expression of Ki‐67 was significantly lower in Groups G4 and G5 compared to the other groups. TUNEL staining (indicating cell apoptosis) exhibited abundant green fluorescence in Groups G4 and G5. HIF‐α staining results indicated that Groups G4 and G5 achieved the most significant inhibitory effects, suggesting that TPZ successfully penetrated the hypoxic regions of the tumor and effectively inhibited the expression of HIF‐α in tumor cells.

Regarding the expression of CD3^+^CD8^+^CD69^+^, the activation of CD8⁺ T cells was significantly higher in Groups G4 and G5 compared to Groups G1 and G2 (Figure 7F). This further demonstrates that the PEM strategy for deep tumor drug delivery effectively activated the activity of CD8^+^ T cells infiltrating the tumor region. Additionally, cytokines in the peripheral blood serum of mice were also detected. As shown in Figure 7H,K, G4 and G5 exhibited the highest expression levels of TNF‐α and IFN‐γ, the expression levels of IL‐6 and IL‐12 were higher in G5 than in G4. This may be due to the effects of TPZ on drug‐resistant tumor cells, which improved the efficacy of Dox on these cells and further enhanced the expression of local cytokines.

In summary, the PEM system, under light irradiation, effectively delivered the hypoxia‐activated drug TPZ to the deep hypoxic regions of the tumor, significantly improving the therapeutic efficacy against the drug‐resistant tumor cell model. The combination of traditional chemotherapeutic drugs with hypoxia‐activated prodrugs further enhanced the therapeutic efficacy against drug‐resistant tumor cells.

## 3 Conclusion

In summary, a PEM‐based drug delivery system has been successfully developed, representing a significant breakthrough in the field of tumor therapy. This system notably improves the efficiency of drug delivery to the deep layers of tumors, thereby enhancing the overall efficacy of antitumor treatment. Notably, the PEM strategy, with its unique mechanism of action, offers a highly promising solution to the intractable problem of MDR caused by tumor hypoxia. The traditional chemotherapeutic drug DOX and the hypoxia‐activated prodrug TPZ are ingeniously encapsulated within thylakoid membranes, which are then integrated into macrophages, endowing them with photo‐empowering capabilities. With this innovative drug delivery strategy, the limitations imposed by the immunosuppressive tumor microenvironment are successfully overcome, bringing new hope to tumor therapy. Under normoxic conditions, Dox exerts significant cytotoxic effects on peripheral tumor cells, effectively inhibiting their growth and proliferation. Upon light stimulation, a photochemical reaction is triggered by the thylakoid, which significantly enhances the mobility of the macrophage and enables PEMD&T to penetrate into the hypoxic regions of the tumor. In these hypoxic areas, TPZ is activated to generate highly toxic free radicals that precisely eliminate hypoxia‐resistant tumor cells. The construction of the PEM system fully exploits the unique biological properties of macrophages and the energy‐generating capabilities of thylakoids, drawing inspiration from the process of photosynthesis in plants. This ingenious design not only greatly improves drug delivery efficiency and penetration depth but also minimizes the interference of the immunosuppressive tumor microenvironment with therapeutic outcomes. The successful development of the PEM strategy provides a novel and effective solution for deep tumor drug delivery and overcoming hypoxia‐induced MDR, paving a hopeful new path for the development of advanced anti‐tumor therapeutic strategies in the future.

## 4 Experimental Section

### Materials

Tirapazamine (TPZ), Doxorubicin hydrochloride (Dox·HCl), Green CMFDA, and CellTracker Blue CMAC were purchased from MedChemExpress (Shanghai, China). The thylakoid membrane extraction kit was obtained from Real‐Times (RTU5002). Oligomycin (Oligo), Sodium dichloroacetate (DCA), and ATP were purchased from TCI (Shanghai, China). HIF‐1α (D1S7W) XP Rabbit mAb (RRID: AB_2 799 095) and XPF antibody (RRID: AB_1 524 575) were purchased from Cell Signaling Technology (USA) and Abcam (USA) respectively. FITC anti‐mouse CD3ε (RRID: AB_312 671), PE anti‐mouse CD8a (RRID: AB_312 747), APC anti‐mouse CD69 (RRID: AB_492 843), Alexa Fluor(R) 700 anti‐mouse CD4 (RRID: AB_493 699), Brilliant Violet 421 anti‐mouse IFN‐γ (RRID: AB_2 563 105), PE anti‐mouse CD25 (RRID: AB_312 856), APC FOXP3 Monoclonal Antibody (RRID: AB_469 457), Brilliant Violet 421 anti‐mouse/human CD44 (RRID: AB_10 895 752), APC anti‐mouse CD62L (RRID: AB_313 099) were acquired from Biolegend (USA).

### Preparation of Micelles@Dox and Micelles@TPZ

For the preparation of Micelles@Dox, a mixed solution of Dox (5 mg) and triethylamine (5 µL) in DMSO (1 mL) was added to a mPEG‐PLA solution (5 mg mL^−1^, in DMF). After stirring the mixture for 2 h under N_2_ protection, the mixture was transferred to a dialysis bag (MWCO: 3500 Da) and dialyzed against deionized water. Finally, the solution was concentrated using an ultrafiltration centrifuge tube (cut‐off: 100 kDa) and stored at 4 °C until use.

Micelles@TPZ were prepared via the thin‐film hydration method. TPZ and mPEG‐PLA were dissolved in a 10 mL mixed solution of chloroform and acetone with thorough mixing. The solution was then transferred to a round‐bottom flask and purified by rotary evaporation to remove the chloroform and acetone. Subsequently, Micelles@TPZ were obtained by slowly injecting deionized water into the round‐bottom flask and stirring vigorously for 10 min.

### Preparation of PEMD and PEMT

Thylakoids were acquired from fresh spinach according to the instructions of the membrane extraction kit (Real‐Times, RTU5002) and extruded 10 times through a 400 nm polycarbonate porous membrane using a liposome extruder (Morgec, LiposoEasy LE‐15). For TK@Dox, a mixed solution of Mi@Dox and thylakoid was extruded 15 times and centrifuged with an ultrafiltration centrifuge tube to condense the chlorophyll concentration to acquire. The TK@Dox at the bottom of the tube was resuspended in pre‐cooled PBS (2% (v/v) penicillin‐streptomycin) and stored at 4 °C. Then, Tk@Dox was added to a 10 cm cell culture dish containing Raw264.7 cells. After incubation for 6 h, the supernatant was removed, and the cells were washed three times with PBS to remove unincorporated TK@Dox for obtaining PEMD. Additionally, the preparation process for PEMT was the same as described above, except that Mi@Dox was replaced with Mi@TPZ.

### Cell Culture and Treatments

Mouse breast carcinoma cells 4T1 (RRID: CVCL_0125), human breast carcinoma cells MCF‐7 (RRID: CVCL_0031), and mouse monocyte‐macrophage cells RAW264.7 (RRID: CVCL_0493) were purchased from Procell Life Science & Technology Co., Ltd. (Wuhan, China) in February 2023. The 4T1‐mCherry cells (RRID: CVCL_C8UZ) were obtained from Viraltherapy Technology Co., Ltd. (Wuhan, China) in September 2024. These cells were cultured in RPMI‐1640 medium (Sigma Aldrich, R6504) supplemented with 10% (v/v) fetal bovine serum (Gibco, 10099141C) and 1% (v/v) penicillin‐streptomycin (Gibco, 15 140 122). The mouse breast adriamycin‐resistant cells 4T1/ADR and human breast adriamycin‐resistant cells MCF‐7/ADR were established with technical support from Viraltherapy Technology Co., Ltd. (Wuhan, China). The parental 4T1 and MCF‐7 cells were subjected to stepwise increases in Dox concentrations (50, 100, 250, 500, 1000, and 2000 ng mL^−1^) for 48 h, followed by 3–5 passages at each concentration to induce drug resistance. The parental cells were defined as Passage 0 (P0), and cells subjected to drug treatment at each passage were designated as P1, P2, P3, and so on. After 30 passages, the drug‐resistant 4T1 cells were maintained in RPMI‐1640 medium containing 1000 ng mL^−1^ Dox.

All the cell lines described above were authenticated via short tandem repeat analysis and confirmed to be contamination‐free.

The cell‐specific motility medium consisted of RPMI 1640 medium, 2 mm glutamine (Gibco, A2916801), 0.5% bovine serum albumin (Gibco, AM2616), and 10 mm HEPES (Gibco, 15 630 080). Prior to cell motility assays, cellular metabolic abnormalities were induced at specific time points using the following metabolic inhibitors: 0.5 µm oligomycin (Sigma Aldrich, 495 455), 10 mm sodium dichloroacetate (TCI, D1719), 0.8 µg mL^−1^ SLO (Sigma Aldrich, SAE0089), and 0.75 mm ATP (TCI, A0157). An anaerobic environment was created using anaerobic gas‐generating bags (MGC, A‐7) and 100% nitrogen gas.

### In Vitro Macrophage Motility Performance Assay and ATP Measurement

RAW264.7 cells were seeded into glass‐bottom 6‐well plates and treated with oligomycin, DCA, thylakoids, and Tk@TPZ (chlorophyll: 0.25 µg mL^−1^, TPZ: 0.03 µg mL^−1^) for 6 h, and then cells were exposed to LED light (660 nm, 4500 lux) for 30 min. Define RAW264.7 cells that engulf thylakoids as PEM, and those that took up Tk@TPZ as PEMT. Subsequently, cells were stained with 1 µm Green CMFDA (MCE, HY‐126561) for 15 min at 37 °C and recorded by CLSM with a 37 °C heated chamber (Olympus FV3000). In the hypoxia experiment, cells with different treatments were placed in a hypoxia box for 8 h before recording with a CLSM. Before supplementing ATP to hypoxic RAW264.7 cells, they needed to be treated with SLO for 1 h. For recording conditions, 60 images (with a 500‐second time interval) were acquired using a 20× objective (Speed: 2 µs pixel^−1^, Scan Size: 800 × 800).

For intracellular ATP detection, the treated cells were harvested and lysed with RIPA buffer for 1 h. An enhanced ATP assay (Beyotime, S0027) was used to measure the ATP content of cell lysates.

### 3D Cell Culture and Penetration Evaluation of PEMD

The 100 µL 3D cell culture plate coating solution (Beyotime, C0365) was added into U‐bottom 96‐well plates and incubated overnight. After that, 4T1 cells were seeded into the pre‐treated 96‐well plates at a density of 1 × 10^3^ cells per well for 48 h. Once the 3D tumor spheroids were constructed, the culture medium was replaced with fresh medium containing Mi@Dox and PEMD. After 6 h of incubation, the tumor spheroids were either irradiated with LED light (660 nm, 0.3 W cm^−2^) for 3 min or left untreated, and then cultured for an additional 12 h. Finally, the tumor spheroids were washed twice with DPBS and observed by CLSM.

### Intravital Imaging Assay with Dorsal Window Chamber 4T1‐mCherry Tumor Model

A dorsal window chamber tumor model was constructed as previously described.^[^ ^38^ ^]^ Briefly, Balb/c mice were anesthetized with isoflurane, and the back hair was removed, followed by the implantation of a titanium frame on the back. One day later, the superficial skin layer within the frame was peeled off and injected with 4T1‐mCherry cells (6 × 10^5^ cells per mouse, 20 µL). Subsequently, a 10 mm‐diameter coverslip was placed and fixed on the peeled skin. After 4 days, the dorsal window mice were intravenously injected with CMAC‐labeled RAW264.7, PEM, and PEMT (5 × 10^6^ cells per mouse, 200 µL). After 12 h, the chamber was exposed to LED light or not (660 nm, 3 mW cm^−2^) for 30 min. Next, 6 hours later, the mice with dorsal window chambers were anesthetized intraperitoneally with 2.5% tribromoethanol (15 µL g^−1^) and immobilized on a homemade warm plate, then placed on the microscope carrier. For tumor 3D image recording conditions, 500 images were acquired using a 10 ×objective (Speed: 2 µs pixel^−1^, Scan Size: 1024 × 1024).

### In Vivo Tumor Accumulation and Deep Penetration

To investigate the distribution and tumor accumulation of PEMT, 4T1 tumor‐bearing mice were established (tumor volume reached ≈200 mm^3^) and randomly divided into two groups that were intravenously injected with free DiR and DiR‐labeled PEMT (at a dosage of 4 × 10^6^ cells per mouse, equal to DiR: 2 mg kg^−1^). At present time points, fluorescence images (ex/em = 745/800 nm) were obtained using the Caliper IVIS animal imaging system. After mice were photographed at 24 h, organs and tumors were excised for FL imaging ex vivo.

To assess tumor‐targeting and accumulation of the macrophage‐based vehicle, female Balb/c mice with 4T1 tumors were intravenously injected with Mi@Dox and PEMD (at a dosage of 4 × 10^6^ cells per mouse, equal to Dox: 0.1 mg kg^−1^). Tumor tissue was harvested at 6, 12, and 24 h post‐injection and ground to obtain a single‐cell suspension, which was then analyzed by flow cytometry (Beckman Coulter, CytoFLEX‐LX) to assess tumor‐targeting.

For evaluating the penetration of PEMD with or without light irradiation, tumor‐bearing female mice were injected with PEMD (at a dosage of 4 × 10^6^ cells per mouse, equal to Dox: 0.1 mg kg^−1^) through the tail vein. The tumor region of mice was exposed to laser irradiation or not (660 nm, 0.3 W cm^−2^) for 5 min at 12 h post PEMD injection. After 12 h, the solid tumor tissues were excised and prepared into frozen sections at different depths for imaging with CLSM.

To determine the effect of light exposure on hypoxic areas of PEMT‐infiltrated tumors, 12 h after DiO‐labeled PEMT (at a dosage of 4 × 10^6^ cells per mouse, equal to TPZ: 0.05 mg kg^−1^) were injected into mice bearing subcutaneous 4T1 tumors via the tail vein, the tumor sites were exposed to laser irradiation or not (660 nm, 0.3 W cm^−2^) for 5 min. The tumor‐bearing mice were injected intraperitoneally with pimonidazole (60 mg kg^−1^) 11 h after the end of light irradiation. One hour later, the mice were euthanized, and the tumor tissues were removed, fixed, embedded, sectioned, and subjected to immunofluorescence staining.

### Orthotopic 4T1 and 4T1‐ADR Tumor Therapeutic Studies with PEMD&T

Female Balb/c mice were subcutaneously injected with 4T1 or 4T1‐ADR cells (1.5 × 10^6^ cells per mouse) at the right thorax to construct the 4T1 tumor model. When the tumor volume reached ≈100 mm^3^ after 8 days, the mice were randomly divided into five groups: (G1) Light, (G2) PEMD, (G3) PEMD&T, (G4) PEMT + Light, and (G5) PEMD &T + Light (dosage: 4 × 10^6^ cells per mouse, equivalent to Dox: 0.1 mg kg^−1^, TPZ: 0.05 mg kg^−1^, Tk: 0.075 mg kg^−1^). Groups G1, G4, and G5 were exposed to laser irradiation (660 nm, 0.3 W cm^−2^) for 5 min at 12 h post‐tail vein injection. The entire treatment procedure was repeated every 5 days for a total of four times, completing the 15‐day treatment. Tumor sizes and body weights were measured every 2 days. At the end of the experiment, all mice were sacrificed, and the tumors were collected, weighed, and photographed. The tumors and major organs were then fixed in 4% paraformaldehyde for pathological analysis via H&E staining. For tumor tissues, HIF‐1α, Ki67, and TUNEL assays were used to detect levels of hypoxia, proliferation, and apoptosis, respectively.

### Flow Cytometry and Serum Cytokines Analysis

Tumors were harvested from 4T1 or 4T1/ADR tumor‐bearing mice in different experimental groups. Single‐cell suspensions were prepared by cutting the tumor tissue into pieces of ≈1 mm^3^ and incubating them in RPMI 1640 medium containing collagenase IV (1 mg mL^−1^), hyaluronidase (0.2 mg mL^−1^), and DNase I (0.2 mg mL^−1^) in a 37 °C‐incubator shaker for 80–90 min. Subsequently, tumor‐infiltrating lymphocytes (TILs) were obtained by filtering the digested tissue through 75 µm cell strainers and then using a lymphocyte separation solution (Solarbio, P9000).

Simultaneously, spleens were gently ground and filtered through 75 µm cell strainers to create single‐cell suspensions. Red blood cells were lysed using RBC lysis buffer (Biolegend, 420 301). For intracellular staining of FOXP3 or IFN‐γ proteins, the True‐Nuclear Transcription Factor Buffer Set (Biolegend, 424 401) and Intracellular Staining Permeabilization Wash Buffer (Biolegend, 421 002) were employed.

For flow cytometric analysis of tumor lymphocytes, cells were first treated with the Zombie Violet Fixable Viability Kit (Biolegend, 423 114) before antibody staining. For T cell activation detection, the following antibodies were used: FITC anti‐CD3 (Biolegend, 100 306), PE anti‐CD8 (Biolegend, 100 708), and APC anti‐CD69 (Biolegend, 104 514).

For analysis of the cytotoxic function of cytotoxic T‐lymphocytes (CTLs), the following reagents and antibodies were used: Brefeldin A (Biolegend, 42 601), FITC anti‐CD3, AF700 anti‐CD4 (Biolegend, 100 430,), PE anti‐CD8, and BV421 anti‐IFN‐γ (Biolegend, 505 830,).

For detection of the Treg cell population, cells were stained with FITC anti‐CD3, AF700 anti‐CD4, PE anti‐CD25 (Biolegend, 102 007,), and APC anti‐FOXP3 (Invitrogen, 17‐5773‐82).

For analysis of effector memory T cells in the spleen, the following antibodies were used: FITC anti‐CD3, AF700 anti‐CD4, PE anti‐CD8, BV421 anti‐CD44 (Biolegend, 103 039), and APC anti‐CD62L (Biolegend, 104 412).

To assess the levels of immunostimulatory factors (TNF‐α, IFN‐γ, IL‐6, and IL‐12p70) in serum, corresponding ELISA kits were used according to the manufacturer's instructions.

### Imaging Processing and Analysis

For velocity analysis of living cell imaging, movies were obtained using Olympus FV3000 and processed using ImageJ to analyze cell movement velocity, movement trajectories, and the movement tracking diagrams were then further generated in Matlab.

For macrophage motility and tissue infiltration analysis via dorsal window intravital imaging, ImageJ was used to process living image videos captured on a confocal microscope to obtain movement velocity and movement trajectory. 3D reconstruction and infiltration analysis were performed with Imaris and ImageJ.

### Ethics Statement

All animal experiments were performed in accordance with the Guide for the Care and Use of Laboratory Animals, and obtained approval from the Experimental Animal Ethics Committee in Hainan University (HNUAUCC‐2023‐0096).

### Statistical Analysis

All data were expressed as mean ± standard deviation (SD) from at least three independent replicate experiments, and the P values were presented as follows: *P < 0.05, **P < 0.01, ***P < 0.001, ****P < 0.0001, ns means not significant. If unspecified, one‐way ANOVA followed by Tukey's test was used for statistical analysis. When comparing two or more groups, statistical analysis of variance was performed using two‐way ANOVA with the Scheffé post hoc test.

## Conflict of Interest

The authors declare no conflict of interest.

## Author Contributions

Z.F., X.C., and S.L. contributed equally to this work.

## Supporting information



Supporting Information

## Data Availability

The data that support the findings of this study are available from the corresponding author upon reasonable request.
