# Supplementary material for: Photo‐Empowered Macrophage‐Based Drug Delivery System Overcomes Motility Suppression and Significantly Enhances Deep Tumor Drug Delivery
Source: Adv Sci (Weinh). 2025 Nov 9;13(5):e15349. doi: 10.1002/advs.202515349 (PMC12850126; doi:10.1002/advs.202515349)
Supplement: Supplementary file 1 — Supporting Information [file ADVS-13-e15349-s001.docx]

Supplementary Materials for

**Photo-Empowered Macrophage-Based Drug Delivery System Overcomes Motility Suppression and Significantly Enhances Deep Tumor Drug Delivery**

Zhaoming Fu^1, 2^, Xin Cui^1, 2^, Shanshan Liu^1, 2^, Lu Gao^1, 2^, Yingang Sun^1, 2^, Yujun Chen^1, 2^, Mianlong Li^1, 2^, Yuxin Fan^1, 2^, Jing Kuang^3^, Wen Song^1, 2, *^, Feifan Zhou^1, 2, *^

^1^ State Key Laboratory of Digital Medical Engineering, School of Biomedical Engineering, Hainan University, Sanya 572025, China

^2^ Key Laboratory of Biomedical Engineering of Hainan Province, One Health Institute, Hainan University, Sanya 572025, China

^3^ Institute of Pathology, Tongji Hospital, Tongji Medical College, Huazhong University of Science and Technology, Wuhan 430030, China

* Correspondence: [zhouff@hainanu.edu.cn](mailto:zhouff@hainanu.edu.cn) (F. Zhou), [songwen@hainanu.edu.cn](mailto:songwen@hainanu.edu.cn) (W. Song)


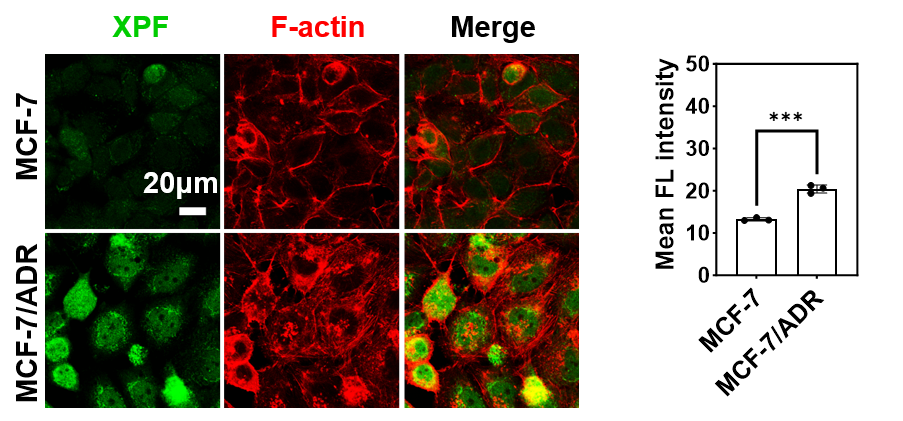


**Figure S1.** Immunofluorescence staining and fluorescence semi-quantitative analysis of XPF protein expression in MCF-7 and MCF-7/ADR cells (n = 3). Data are presented as mean ± SD. Statistical significance was calculated via one-way ANOVA with Tukey’ post hoc test. (**p* < 0.05, ***p* < 0.01, ****p* < 0.001).


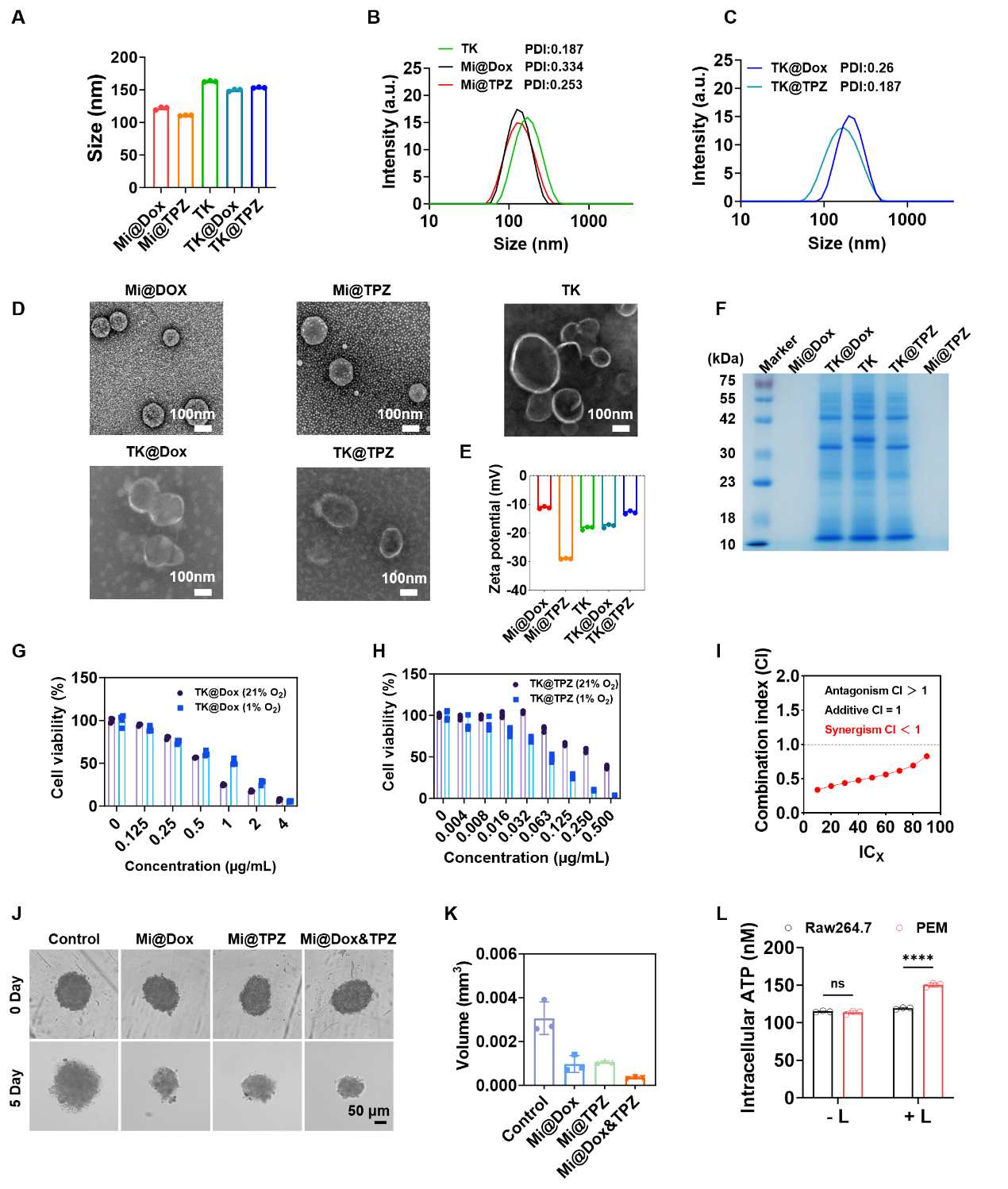


**Figure S2.** **(A)** Average hydrodynamic diameters of Mi@Dox, Mi@TPZ, Tk, Tk@Dox and Tk@TPZ (n = 3). Hydrodynamic diameters distributions of **(B)** Mi@Dox, Mi@TPZ, Tk and **(C)** Tk@Dox, Tk@TPZ. **(D)**TEM image of Mi@Dox, Mi@TPZ, Tk, Tk@Dox and Tk@TPZ. **(E)** Zeta potential of Mi@Dox, Mi@TPZ, Tk, Tk@Dox and Tk@TPZ (n = 3). **(F)** SDS-PAGE protein analysis of Tk, Mi@Dox, Tk@Dox, Tk, Mi@TPZ and Tk@TPZ. Cell viability of **(G)** Tk@Dox and **(H)** Tk@TPZ treated 4T1 under 21% or 1% O_2_ (n = 3). **(I)** Combination Index of Tk@Dox and Tk@TPZ against 4T1 cells. **(J)** Optical microscopy images of 3D tumor spheroids before and after treatment with various drugs. **(K)** Quantitative analysis of the volume of 3D tumor spheroids after five days of treatment (n = 3). **(L)** Average intracellular ATP content of Raw264.7 cells and PEM with light or not (n = 3). Data are presented as mean ± SD. Statistical significance was calculated via one-way ANOVA with Tukey’ post hoc test. (**p* < 0.05, ***p* < 0.01, ****p* < 0.001, *****p* < 0.0001).


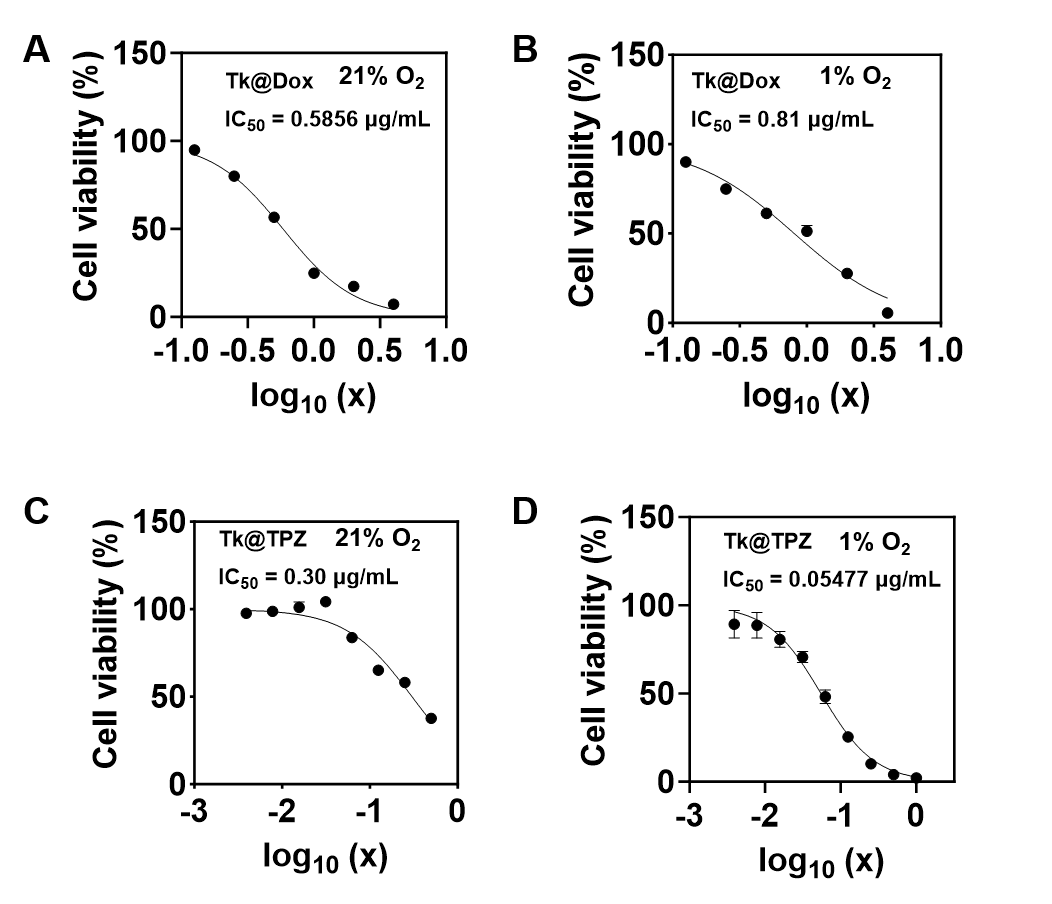


**Figure S3.** *In vitro* 4T1 cells viability treated with Tk@Dox **(A)** in 21% O_2_ (Normoxia) condition and **(B)** in 1% O_2_ (Hypoxia) condition. *In vitro* 4T1 cells viability treated with Tk@TPZ **(C)** in 21% O_2_ (Normoxia) condition and **(D)** 1% O_2_ (Hypoxia) condition (n = 3). Data are presented as mean ± SD.


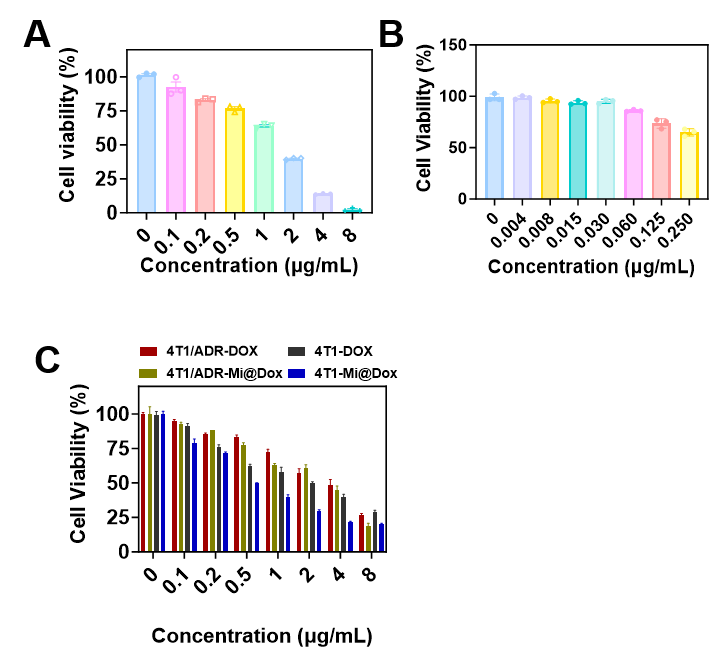


**Figure S4.** *In vitro* cell viability of Raw264.7 cells incubated with **(A)** Mi@Dox and **(B)** Mi@TPZ (n = 3). **(C)** Cell viability following treatment of 4T1 and 4T1/ADR with DOX and Mi@Dox (n = 3). Data are presented as mean ± SD.


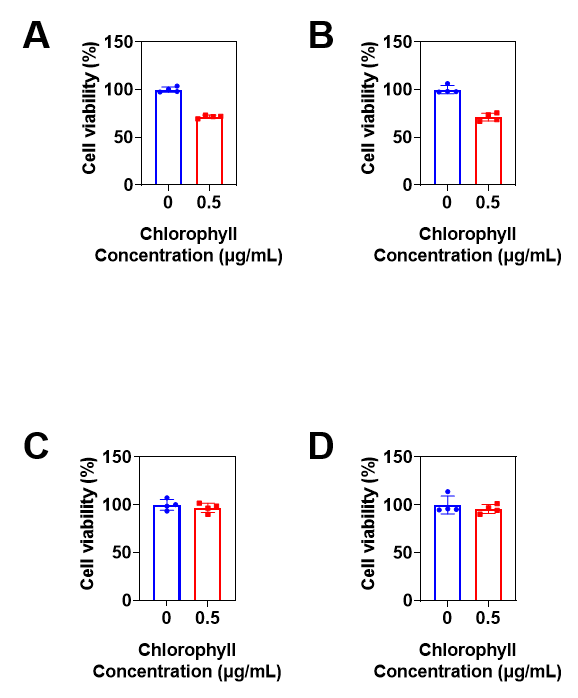


**Figure S5.** *In vitro* cell viability of Raw264.7 cells incubated with Tk@Dox, treated **(A)** with or **(B)** without light irradiation (n = 4). In vitro cell viability of Raw264.7 cells incubated with Tk@TPZ, treated **(C)** with or **(D)** without light irradiation (n = 4). Data are presented as mean ± SD.


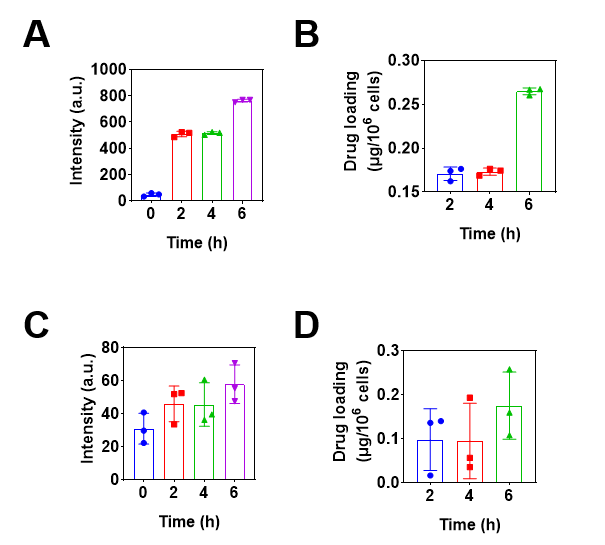


**Figure S6.** **(A)** Fluorescence intensity of Dox in PEMD and the **(B)** corresponding drug loading at different incubation times (n = 3). **(C)** Fluorescence intensity of TPZ in PEMT and the **(D)** corresponding drug loading at different incubation times (n = 3). Data are presented as mean ± SD.


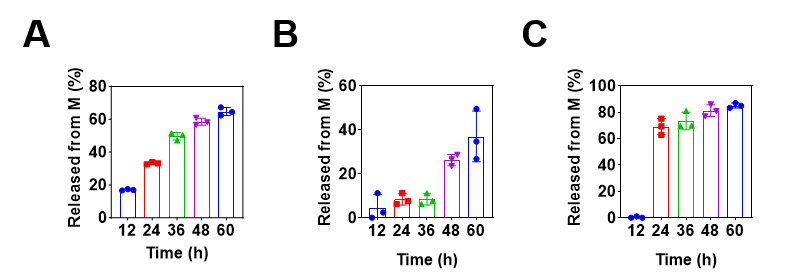


**Figure S7.** **(A)** Drug release profiles of PEMD at different time points under normoxic conditions (21% O₂) (n = 3). **(B)** Drug release profiles of PEMT at different time points under normoxic conditions (21% O₂) (n = 3). **(C)** Drug release profiles of PEMT at different time points under hypoxic conditions (1% O₂) (n = 3). Data are presented as mean ± SD.


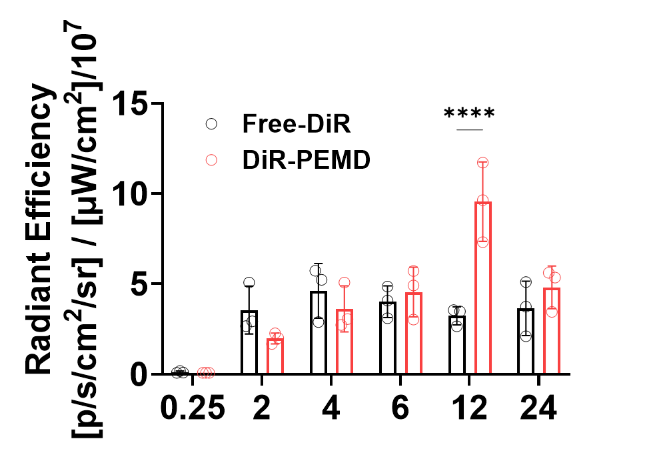


**Figure S8.** *In vivo* semi-quantitative analysis of radiant efficiency at tumor region with various time point (n = 3). Data are presented as mean ± SD. Statistical significance was calculated via one-way ANOVA with Tukey’ post hoc test. (**p* < 0.05, ***p* < 0.01, ****p* < 0.001, *****p* < 0.0001).


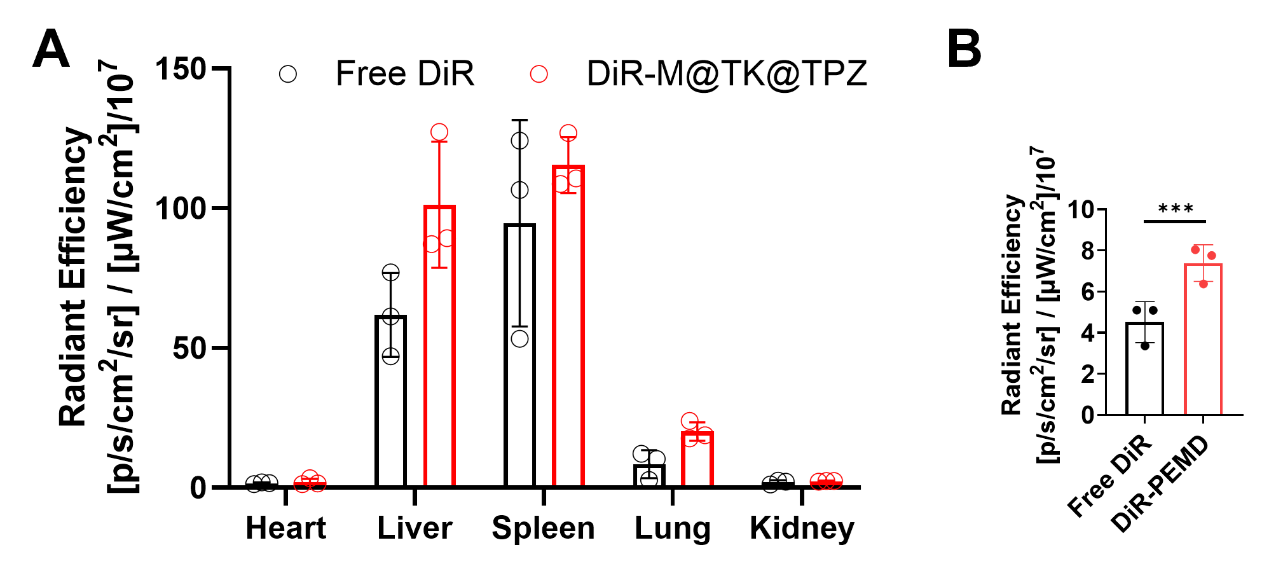


**Figure S9.** Semi-quantitative analysis of radiant efficiency with **(A)** isolated major organs and **(B)** tumor tissues at 24 hours post-intravenous injection (n = 3). Data are presented as mean ± SD. Statistical significance was calculated via one-way ANOVA with Tukey’ post hoc test. (**p* < 0.05, ***p* < 0.01, ****p* < 0.001).


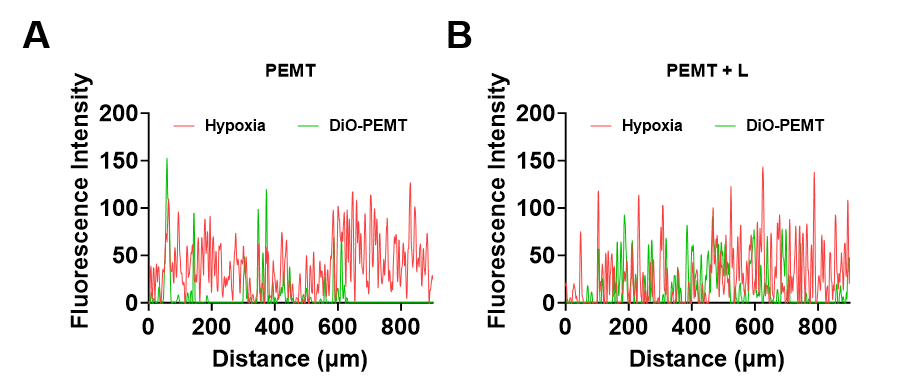


**Figure S10.** DiO-labeled PEMT (green fluorescence) and pimonidazole-labeled hypoxic regions (red fluorescence) intensity in the red line marked area of tumor sections treated **(A)** with or **(B)** without light irradiation.


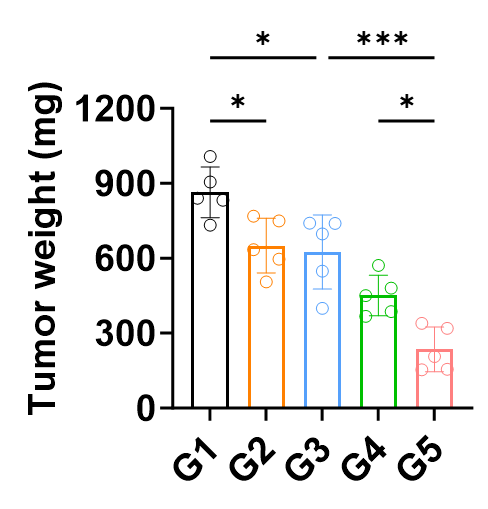


**Figure S11.** Tumor weight in 4T1 tumor-bearing mice at the end of the 16-day treatment period (n = 5). Data are presented as mean ± SD. Statistical significance was calculated via one-way ANOVA with Tukey’ post hoc test. (**p* < 0.05, ***p* < 0.01, ****p* < 0.001).


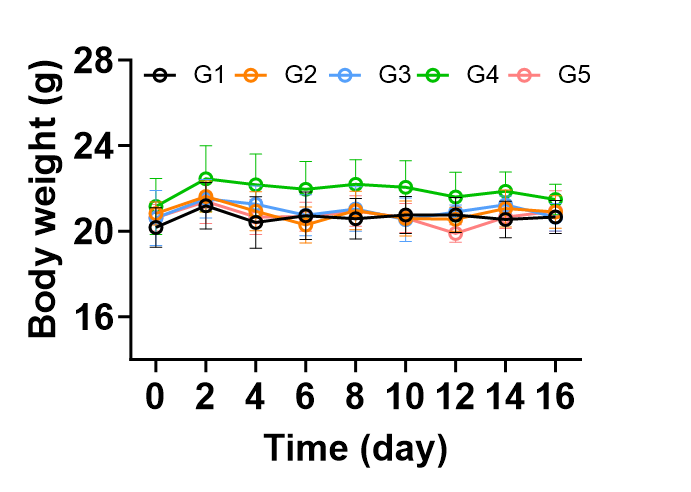


**Figure S12.** Body weight changes of 4T1 tumor-bearing mice over a 16-day treatment period in different groups (n = 5). Data are presented as mean ± SD. Statistical significance was calculated via one-way ANOVA with Tukey’ post hoc test.


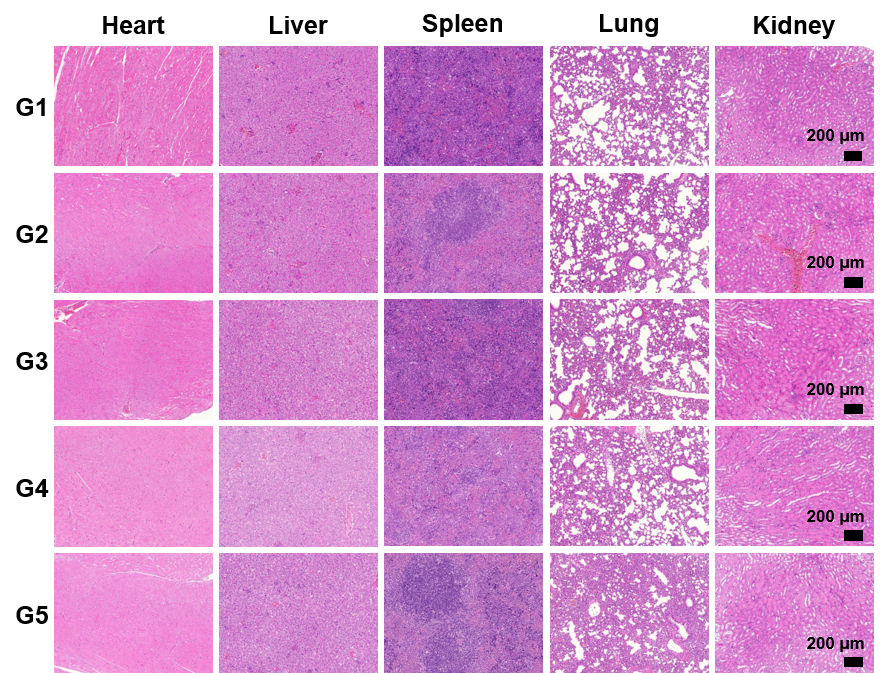


**Figure S13.** H&E staining sections of major tissues (heart, liver, spleen, lung, kidney) from 4T1 tumor-bearing mice at the end of the 16-day treatment period.


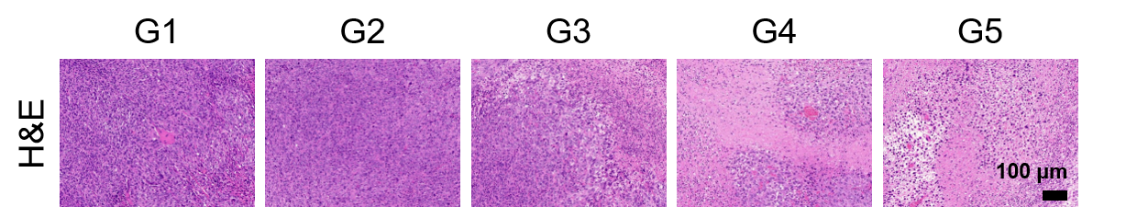


**Figure S14.** H&E staining sections of tumor tissues from 4T1 tumor-bearing mice at the end of the 16-day treatment period.


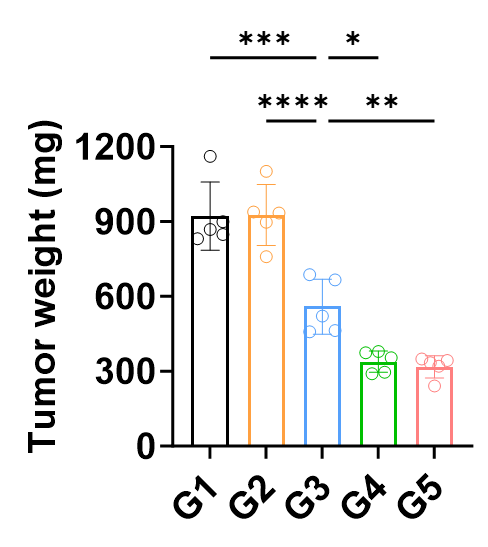


**Figure S15.** Tumor weight in 4T1/ADR tumor-bearing mice at the end of the 16-day treatment period (n = 5). Data are presented as mean ± SD. Statistical significance was calculated via one-way ANOVA with Tukey’ post hoc test. (**p* < 0.05, ***p* < 0.01, ****p* < 0.001, *****p* < 0.0001).


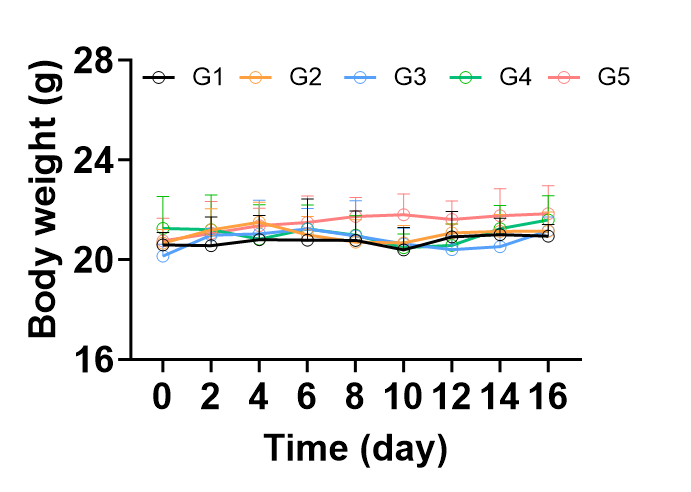


**Figure S16.** Body weight changes of 4T1/ADR tumor-bearing mice over a 16-day treatment period in different groups (n = 5). Data are presented as mean ± SD. Statistical significance was calculated via one-way ANOVA with Tukey’ post hoc test.


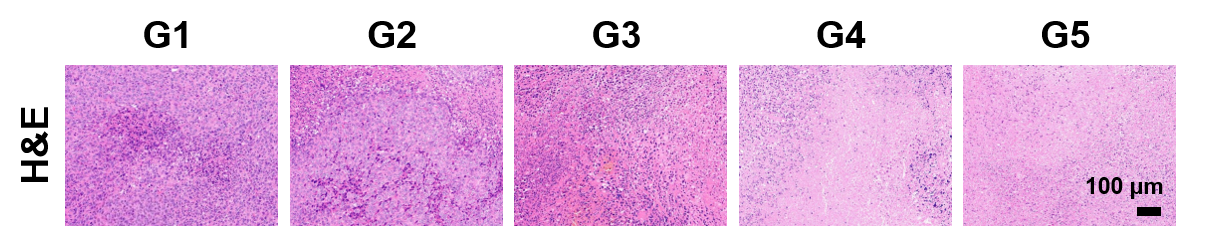


**Figure S17.** H&E staining sections of tumor tissues from 4T1/ADR tumor-bearing mice at the end of the 16-day treatment period.
